# No short-term effect of sinking microplastics on heterotrophy or sediment clearing in the tropical coral *Stylophora pistillata*

**DOI:** 10.1038/s41598-022-05420-7

**Published:** 2022-01-27

**Authors:** Sonia Bejarano, Valeska Diemel, Anna Feuring, Mattia Ghilardi, Tilmann Harder

**Affiliations:** 1 Reef Systems Research Group, Leibniz Centre for Tropical Marine Research (ZMT), Fahrenheitstraße 6, 28359 Bremen, Germany; 2 Department of Marine Ecology, Faculty of Biology and Chemistry, University of Bremen, Leobener Straße 6, 28359 Bremen, Germany; 3 Bund Für Umwelt Und Naturschutz (BUND) E.V., Am Dobben 44, 28203 Bremen, Germany; 4 Biological Oceanography Department, Leibniz Institute for Baltic Sea Research Warnemünde, Seestraße 15, D-18119 Rostock, Germany; 5 Alfred Wegener Institute, Helmholtz Centre for Polar and Marine Research, 27570 Bremerhaven, Germany

**Keywords:** Ecology, Ocean sciences

## Abstract

Investigations of encounters between corals and microplastics have, to date, used particle concentrations that are several orders of magnitude above environmentally relevant levels. Here we investigate whether concentrations closer to values reported in tropical coral reefs affect sediment shedding and heterotrophy in reef-building corals. We show that single-pulse microplastic deposition elicits significantly more coral polyp retraction than comparable amounts of calcareous sediments. When deposited separately from sediments, microplastics remain longer on corals than sediments, through stronger adhesion and longer periods of examination by the coral polyps. Contamination of sediments with microplastics does not retard corals’ sediment clearing rates. Rather, sediments speed-up microplastic shedding, possibly affecting its electrostatic behaviour. Heterotrophy rates are three times higher than microplastic ingestion rates when corals encounter microzooplankton (*Artemia salina* cysts) and microplastics separately. Exposed to cysts-microplastic combinations, corals feed preferentially on cysts regardless of microplastic concentration. Chronic-exposure experiments should test whether our conclusions hold true under environmental conditions typical of inshore marginal coral reefs.

## Introduction

Ocean pollution is a recognised hallmark of the Anthropocene. Marine coastal systems are increasingly confronted with sewage, nutrients, sediments, persistent organic pollutants, hydrocarbons, heavy metals, litter, and radionuclides^1^. Albeit arguably not the most toxic of contaminants, microplastics (0.05–5 mm)^2^ are permanent and frequently encountered by marine biota with adverse consequences^3,4^. With 368 million tons of plastics produced globally every year, > 12 million of them entering the oceans, and 92% made up of microplastics^5,6^, understanding the threats posed by microplastics to marine biota is a global research priority^7^.

Whether the consequences of the interactions between marine biota and microplastics are overstated or warrant social and political action remains debated^8,9^. This is at least partially due to the paucity of experiments using ecologically realistic microplastic concentrations to test whether organisms react similarly to these and natural particles, and whether key ecological functions are impaired^9^. Knowledge on how microplastics affect ecosystem functions in the ocean (e.g. nutrient cycling, herbivory) has lagged behind that of physiological responses of organisms and comes mainly from temperate taxa e.g.^10^. The extent to which microplastics pose a serious threat to key marine tropical ecosystem engineers (e.g. reef-building corals) thus remains incompletely resolved. With several tropical countries accounting for some of the highest quantities of plastic entering the ocean through rivers^11^, and costly actions called for by international, regional, and national political agendas, contributing solid empirical evidence to the debate is paramount.

Tropical reef-building corals are severely threatened by ocean acidification, heat waves and insufficient time windows for recovery, but also exposed to land-based pollution and plastic debris^12,13^. At least more than a quarter of the world’s tropical coral reefs are indeed threatened by watershed-based pollution^14^. It follows that suspended sediment particles are ubiquitous features of corals reefs and likely co-occur with respectable amounts of microplastics. Microplastic concentrations in seawater on tropical coral reefs reportedly range from 0.0001 particles L^−1^ in the South China Sea to 820 particles L^−1^ in Indonesia^15,16^. Suspended microplastics aggregate and interact with other suspended particles (e.g., microalgae, bacteria, and fungi), are biofouled, and thus become denser and sink into coral reef sediments^17,18^. Coral reef sediments can therefore contain up to 48-820 microplastic particles kg^−1^^16,19^.

Whether terrigenous (i.e. from rivers or coastal dredging) or reefal (i.e. originating from biotic and hydrodynamic erosion of calcium carbonate), sediment particles deposit, enter biologically-mediated fluxes, and can be resuspended and redeposited daily^20,21^. Although corals have the ability to feed on sediments and digest their organic fraction^22^, sediments can have sublethal or lethal effects when deposited on corals^23^. These effects are reportedly aggravated by sediment-associated contaminants such as heavy metals, pesticides, toxic sewage substances, and antifoulant paint from ship groundings^24,25^, but less is known about the effects of microplastic-contaminated sediments on corals. In addition to passive sediment-shedding strategies which are a function of colony morphology, fine skeletal architecture, turbulence, and gravity^26^, corals rely on a plethora of active sediment rejection mechanisms. These include tentacle and ciliary movements, contractions, polyp projection, stomodeal distention, hydrostatic inflation, mucus entrapment, movement by mesenterial filaments and capture by nematocysts^23,26,27^, some of which imply high metabolic costs^27^.

Corals have been exposed to a wide range of microplastic concentrations (2.5–9 × 10^10^ particles L^−1^) in the laboratory. Under high microplastic concentrations corals exhibit a variety of responses including active ingestion^28,29^, depleted energy reserves^30^, decreased enzymatic activity, skeleton mineralization rates, growth and heterotrophic feeding rates^31–33^, altered photobiology and oxidative stress^34,35^, polyp mortality^36^, and tissue necrosis and bleaching^37^. Studies using moderate microplastic concentrations report no effect on calcification rate^38^, mild species-specific effects on energy reserves and photosynthetic performance^39,40^, and minor infrequent cumulative effects on tolerance to heat stress^35^. Importantly, both laboratory experiment and field surveys demonstrate that microplastics adhere passively yet copiously to coral surfaces likely tracking differences in coral morphology^41–43^. Of the 20 coral-microplastic experiments published to date, less than half exposed corals to microplastics in combination with other common seawater particulates (e.g. microinvertebrates and sediments)^29,36,41,44^. Only one study so far compared how corals react to both sediments and microplastics, briefly documenting that significantly more cnidocytes are fired towards microplastics^29^ and no study has addressed whether microplastics compromise sediment rejection efficiency in corals.

Heterotrophy (i.e. feeding on suspended zooplankton, phytoplankton, and dissolved organic matter^22^) accounts for up to 66% of the fixed carbon incorporated into coral skeletons, meeting 15–35% of daily metabolic demands of healthy corals and up to 100% of bleached corals^45^. Comparisons between heterotrophy and microplastic ingestion rates are scarce for tropical corals^41,42^, and findings are contradictory. Exposed to polyethylene (PE) microbeads (0.1–0.4 g L^−1^) and natural foods (i.e. *Artemia salina* cysts), the temperate coral *Astrangia poculata* fed preferentially on the microbeads, whereas the tropical single-polyp mushroom coral *Danafungia scruposa* fed tree times more often on *A. salina*^36,41^*.* Four Red Sea coral species ingested and retained microplastics independently of the presence of natural foods^42^. Further, PE microbeads had no effect on heterotrophy rates of the cold water coral *Lophelia pertusa*^32^. As a generalisation regarding the impact of microplastics in natural heterotrophy remains elusive, it is important to investigate this using a range of moderate microplastic concentrations.

**Figure 1:**
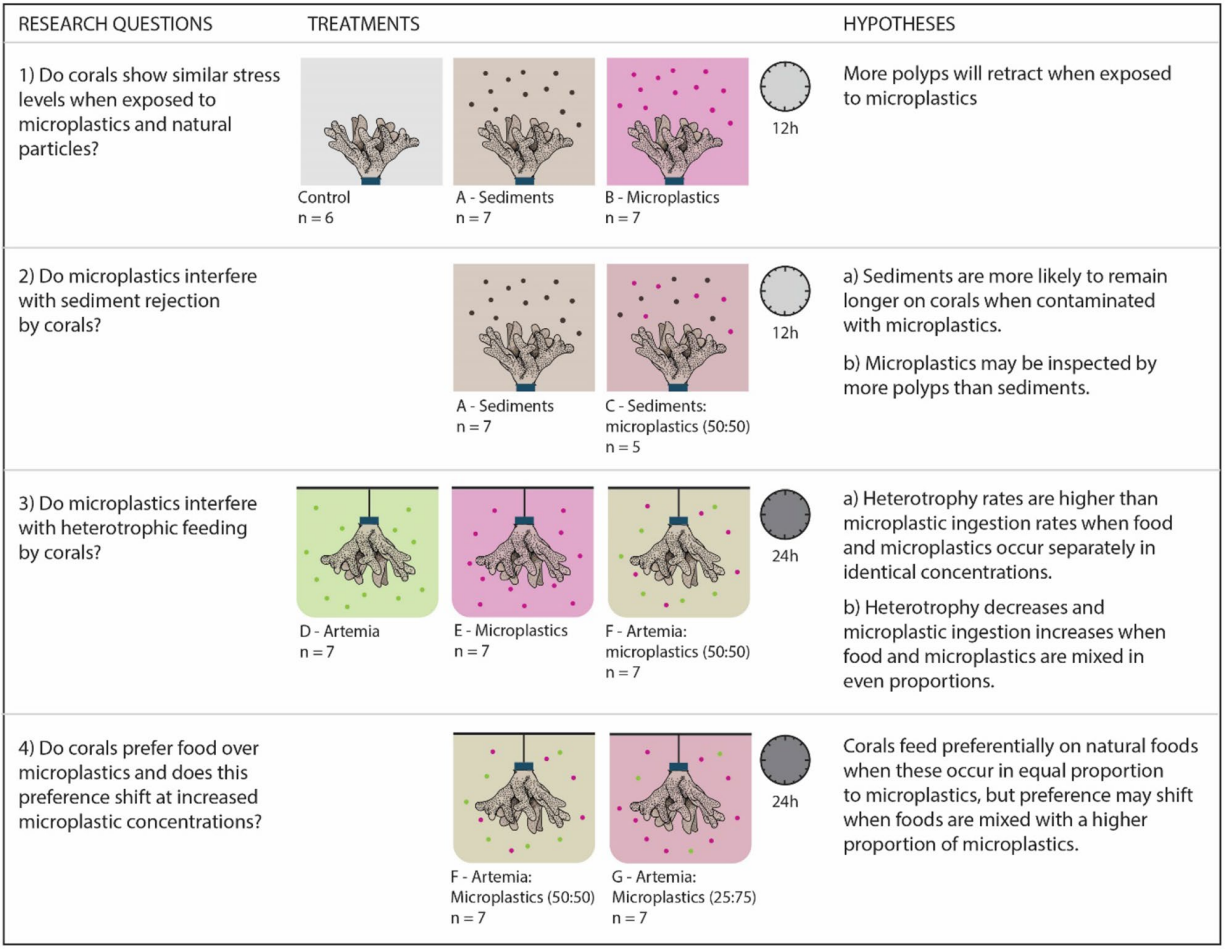
Experimental design of this study. Simplified diagram of the experimental design indicating the treatments compared to resolve four hypothesis-driven research questions. The number of replicates per treatment (n), as well as the duration of each experiment (in hours), are also indicated. All particles were of similar size range (sediments: 256.9 ± 24.3 μm, microplastics: 261.35 ± 27.5 μm, and *A. salina* cysts: 252.08 ± 21.3 μm). Particle concentrations per volume of water of the experimental tank are as follows: Treatment *A*: 2.64 (± 0.33) sediment particles L^−1^, treatment *B*: 2.86 (± 0.41) microplastics L^−1^, treatment *C*: 1.68 (± 0.16) microplastics L^−1^ and 1.45 (± 0.09) sediment particles L^−1^, treatment *D* 100 cysts L^−1^, treatment *E*: 100 microplastics L^−1^, treatment *F*: 50 cysts L^−1^ and 50 microplastics L^−1^, treatment *G*: 25 cysts L^−1^ and 50 microplastics L^−1^.

Here, we examined whether moderate microplastic concentrations usually detected in reef seawater and within sediments (Supplementary Tables S1, S2) affect two key functions of the reef building coral *Stylophora pistillata*, namely sediment shedding and heterotrophic feeding. Using controlled aquaria experiments (Fig. 1), we compared the behavioural response of corals to either reefal sediments or irregular polyethylene terephthalate (PET) microplastics (over 12 h). We then quantified the hourly probability of sediments remaining on corals as a proxy for sediment rejection efficiency in corals exposed to uncontaminated reefal sediments and 50:50 mixtures of reefal sediments and microplastics. Lastly, we measured the heterotrophic feeding rates of corals exposed to uncontaminated samples of *A. salina* cysts and varying ratios of *A. salina*:microplastic combinations as well as feeding selectivity to assess the impact of microplastics on coral feeding function. Macroplastics reportedly increase the susceptibility of tropical corals to disease^13^, but less is known about whether their vital functions are threatened by microplastics. This study is thus a key and timely contribution towards grasping the severity of the impact of plastic pollution on coral reefs and addressing one of the future grand challenges in marine ecosystems^46^.

## Results

### Polyp retraction in response to nonedible particles

**Figure 2:**
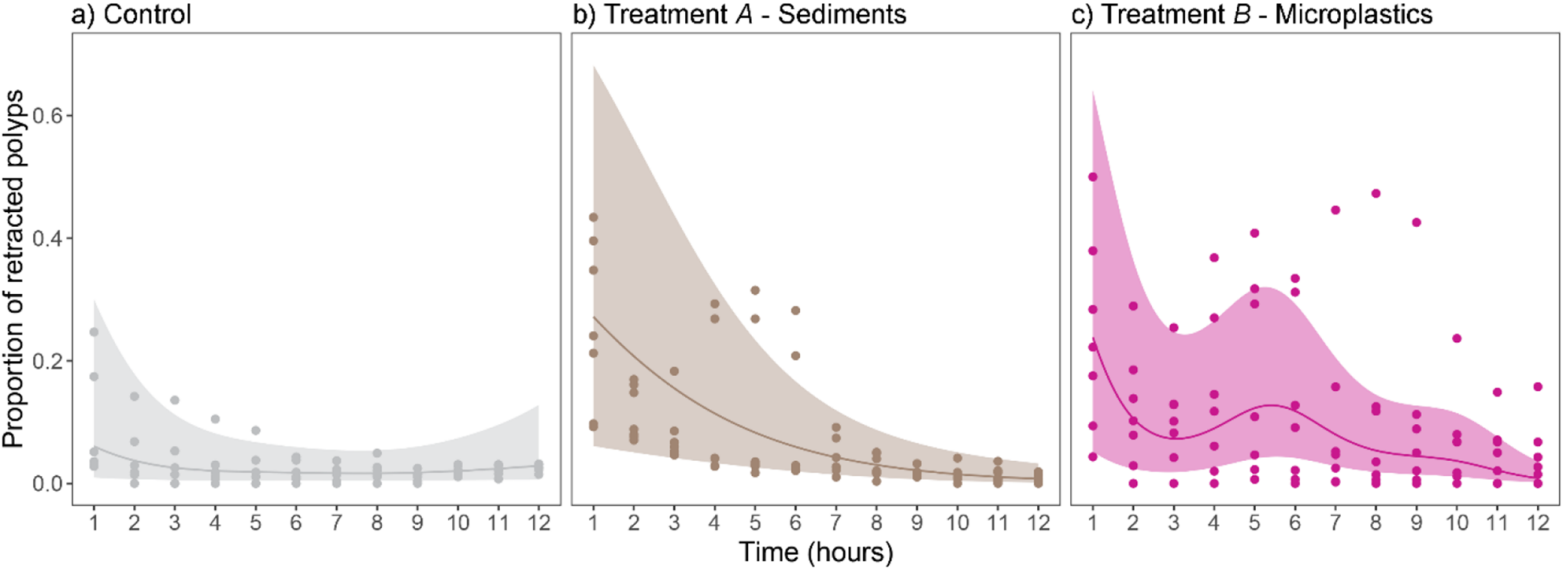
Polyp retraction in response to reef sediments and PET microplastics. Proportion of retracted polyps recorded hourly for 12 h in *S. pistillata* colonies exposed to (**a**) the procedural control, (**b**) reef sediments (treatment *A*), and (**c**) irregular PET microplastics (treatment *B*). Smoothers differed significantly among treatments (Table 1).

When exposed to sediments (treatment *A*), microplastics (treatment *B*), and the procedural control, corals showed different proportions of retracted polyps over time (12 h). Under control conditions, the proportion of retracted polyps remained low (< 0.25). Compared to the control conditions, both sediments and microplastics elicited almost double the proportion of retracted polyps during the first hour of the experiment. In the presence of sediments, polyp retraction decreased gradually, whereas with microplastics polyp retraction changed nonlinearly over time (Fig. 2, Table 1). When exposed to either sediments or microplastics, corals often released thick mucus strains and, less frequently, gas bubbles. Both sediments and microplastics were thus often trapped inside the mucus strains. Some of these mucus strains sloughed off swiftly from the coral fragments, whereas others remained attached to the corals for at least 12 h. Gas bubbles, not exclusively, yet more often produced by corals exposed to particles compared to control conditions, also trapped both sediments and microplastics and removed them from the corals more immediately as these floated up (Supplementary Fig. S1g, h).

**Table 1:** Statistical outputs of models fitted to answer the four research questions of this study.

Outputs of statistical models				
(1) Do corals show similar responses to microplastics and sediments?				
	Estimate	SE	z value	Pr ( >|z|)
*GAMM for proportion data (Family: Binomial)*				
Parametric coefficients				
Intercept (control)	− 3.5676	0.6182	− 5.771	7.88e−9
Treatment *A*	0.7030	0.6871	1.023	0.306
Treatment *B*	0.9009	0.7000	1.287	0.198

	Edf	Ref.df	Chi.sq	P value
*Approximate significance of smooth terms*				
s(*hour*):control	3.904	3.904	59.371	3.69e−12
s(*hour*):treatment *A*	1.000	1.000	9.849	0.0017
s(*hour*):treatment *B*	6.865	6.865	300.271	< 2e−16

(2) Do microplastics interfere with corals’ sediment rejection rates?				
	Coefficient	Robust SE	z value	Pr ( >|z|)
*(a) Residence time*				
Kaplan–Meier curves and Cox proportional hazard model				
Treatments (*A* vs. *B*)	− 0.5236	0.0719	− 7.28	3.35e−13
Treatments (*A* vs. *C*)	0.1708	0.0949	1.80	0.0718
Treatments (*B* vs. *C*)	0.3693	0.1005	3.675	0.0002

	Estimate	SE	95% CI (L)	95% CI (U)
*(b) Number of polyps touched*				
Poisson Hurdle models				
Intercept (Treatment *A*)	− 2.31	0.58	− 3.46	− 1.19
Treatment *B*	1.28	0.37	0.59	2.04
Intercept (Treatment *A*)	− 0.41	0.96	− 2.25	1.56
Treatment *C*	0.51	0.56	− 0.65	1.57
Intercept (Treatment *B*)	− 1.13	0.44	− 2.00	− 0.29
Treatment *C*	− 0.01	0.27	− 0.54	0.50

(3) Do microplastics interfere with corals’ heterotrophic feeding				
	Coefficient	SE	t value	Pr ( >|t|)
*GLS (allowing for heteroscedasticity across treatments) comparing overall feeding rates*				
Intercept (Treatment *D*)	0.9051	0.0297	30.4850	0.0000
Treatment *E*	− 0.6387	0.0324	− 19.7193	0.0000
Treatment *F*	0.1707	0.0633	2.6954	0.0148

(4) Do corals prefer microzooplankton over microplastics?				
	Manly’s *α*	SD	Statistic	P
*T-test for differences between Manly’s α for A. salina and value expected for no selectivity* (0.50)				
Treatment *F*	0.527	0.008	8.38	0.0002
Treatment *G*	0.691	0.026	19.1	0.0000
*T-test for differences between Manly’s α for microplastics and value expected for no selectivity* (0.50)				
Treatment *F*	0.473	0.008	− 8.38	0.0002
Treatment *G*	0.309	0.026	− 19.1	0.0000

	Estimate		Statistic	P
*Welch’s test for difference in Manly’s α for A. salina between treatments*				
Treatments (*F* vs. *G*)	− 0.164		− 15.63	7.87e−7

Estimates of Poisson–Hurdle models are presented on a logarithmic scale.

Significant values are underlined.

### Probability of microplastics and sediments remaining on corals

**Figure 3:**
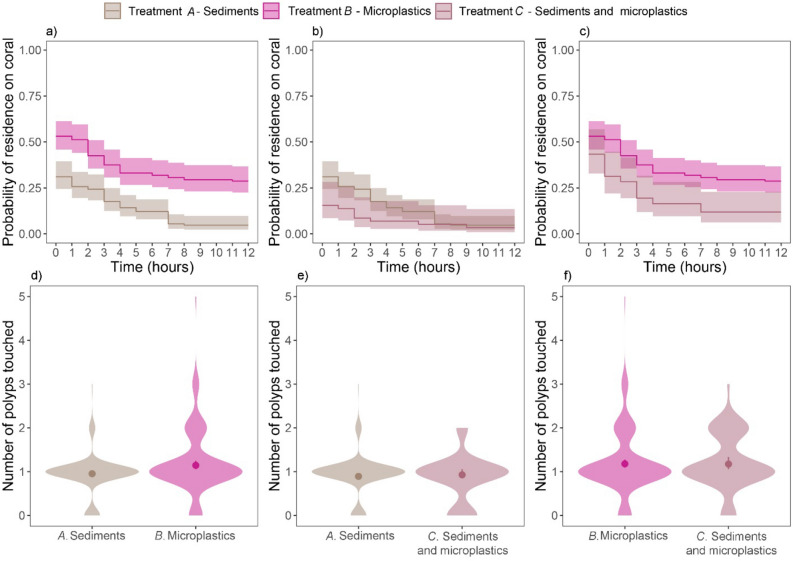
Residence time and polyp contacts of microplastics and sediments on corals. (**a**–**c**) Kaplan–Meier curves (± 95% CIs) indicating the hourly probability of residence on coral tissues of (**a**) microplastics and sediments, when added separately to corals (treatment *A* vs. *B*), (**b**) sediments when these are presented alone (treatment *A*) or within 50:50 mixtures with microplastics (treatment *C*), and (**c**) microplastics when presented alone (treatment *B*) or within 50:50 mixtures with sediments (treatment *C*), for the duration of the experiment. (**d**–**f**) Number of polyps touched by (**d**) microplastics and sediments when added separately (treatment *A* vs. *B*), (**e**) sediments when added alone (treatment *A*) or in 50:50 mixtures with microplastics (treatment *C*), and (**f**) microplastics when added alone (treatment *B*) or in 50:50 mixtures with sediments, for the duration of the experiment. (**d**–**f**) Solid dots represent the fitted values of Bayesian Hurdle Poisson models (± 95% CIs), and violin plots represent the distribution of observed values.

Microplastics had a significantly higher probability of remaining on coral surfaces over time than sediments when added separately onto the corals (Fig. 3a, Table 1). An hour after separate particle addition, the probability of microplastics remaining on corals was 41% higher than that of sediments (Fig. 3a). Over the following seven hours, the probability of adhering to the coral decreased over time in a similar manner for both microplastics and sediments (Fig. 3a). However, for the last five hours of the experiment, the probability of being retained on the coral surface remained ~ 83% higher for microplastics than for sediments (Fig. 3a).

The presence of microplastics within reef sediments did, however, not retard the corals’ efficiency to reject sediments from their surface (Fig. 3b, Table 1). This was evident because the probability of sediment particles remaining on the coral surface decreased similarly, and remained similarly low, regardless of whether they occurred alone or in combination with microplastics (Fig. 3b, Table 1). Interestingly, the probability of residence of microplastics on corals over time was slightly, yet significantly, lower when mixed with sediments than when added alone (Fig. 3c, Table 1).

When added separately, microplastics touched slightly more polyps than an average reefal sediment particle (1.14 (1.05–1.25) vs. 0.95 (0.90–1.02) mean and 95% CI, Fig. 3d and Supplementary Fig. S2, Table 1).

The number of polyps touched by an average sediment particle was similar, regardless of whether it was added separately to the coral or in combination with microplastics (Fig. 3e and Supplementary Fig. S3, Table 1). Similarly, the number of polyps touched by a microplastic was similar regardless of whether it was added separately to the coral or mixed with sediments (Figs. 3f and Supplementary Fig. S4, Table 1).

### Coral heterotrophic feeding rate and microplastic ingestion

**Figure 4:**
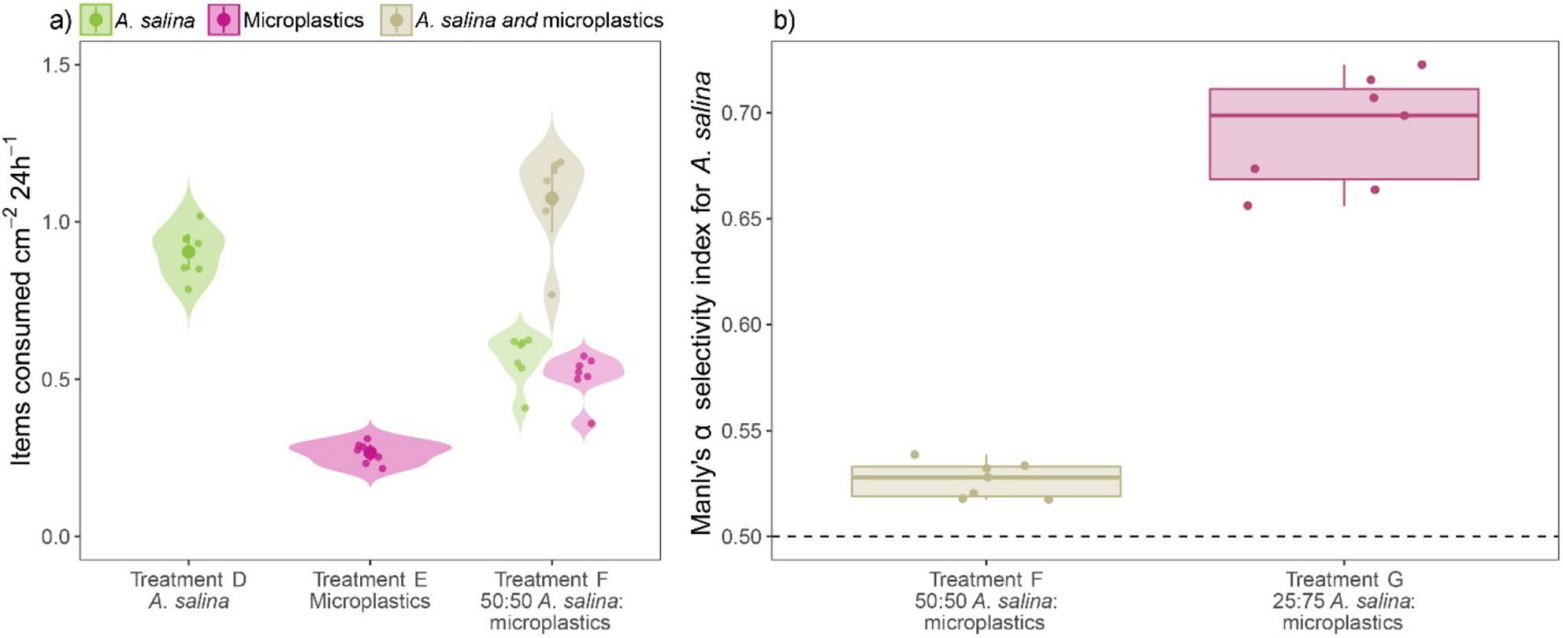
Coral heterotrophy and microplastic ingestion. (**a**) Coral feeding rates (items consumed cm^−2^ 24 h^−1^) per particle type and treatment compared among treatments *D*, *E*, and *F*. Solid (larger) dots represent fitted values of GLS models (± 95% CIs), and violin plots represent the distribution of observed values which are also added as jittered (smaller) dots. (**b**) Manly’s α feeding selectivity index of corals for *A. salina* when exposed to 50:50 and 25:75 *A. salina*:microplastic mixtures (treatments *F* and *G*). The dashed line (Manly’s α = 0.5) represents no selectivity (i.e., prey is used according to availability).

Exposed separately to identical concentrations of *A. salina* cysts and microplastics (i.e. 100 items L^−1^, treatments *D* vs. E), *S. pistillata* ingested three times more cysts than microplastics (p < 0.0001, Fig. 4a, Table 1). Overall coral feeding rates were significantly higher when exposed to even mixtures of cysts and microplastics at 50 items L^−1^ each, than when exposed separately to either particle type (p < 0.0001, Fig. 4a, Table 1). This difference, however, reflects mainly that corals fed almost four times more on microplastics when these occurred at 50 microplastics L^−1^ and in combination with food, than when they occurred alone at 100 microplastics L^−1^ (p < 0.001, Fig. 4a, Supplementary Table S4).

When exposed to even mixtures of *A. salina* cysts and microplastics each at 50 items L^−1^ (treatment *F*), corals ingested similar amounts of each particle type (i.e. 0.56 cysts cm^−2^ 24 h^−1^ and 0.51 microplastics cm^−2^ 24 h^−1^, Fig. 4a). However, corals fed preferentially on *A. salina* cysts (Manly’s α = 0.527 ± 0.008, Fig. 4b, Table 1) while avoiding microplastics (Manly’s α = 0.473 ± 0.008, Table 1). Corals fed selectively on *A. salina* and avoided microplastics also when exposed to *A. salina* at 25 items L^−1^ mixed with 75 microplastics L^−1^ (treatment *G*) (Manly’s α ≠ 0.5, p < 0.001). Indeed, the degree of feeding selectivity for *A. salina* was significantly higher when corals were exposed to uneven 25:75 *A. salina*:microplastic mixtures (treatment *G*) than when exposed to even 50:50 *A. salina*:microplastic mixtures (treatment *F*) (p < 0.001, Fig. 4b, Table 1).

## Discussion

**Figure 5:**
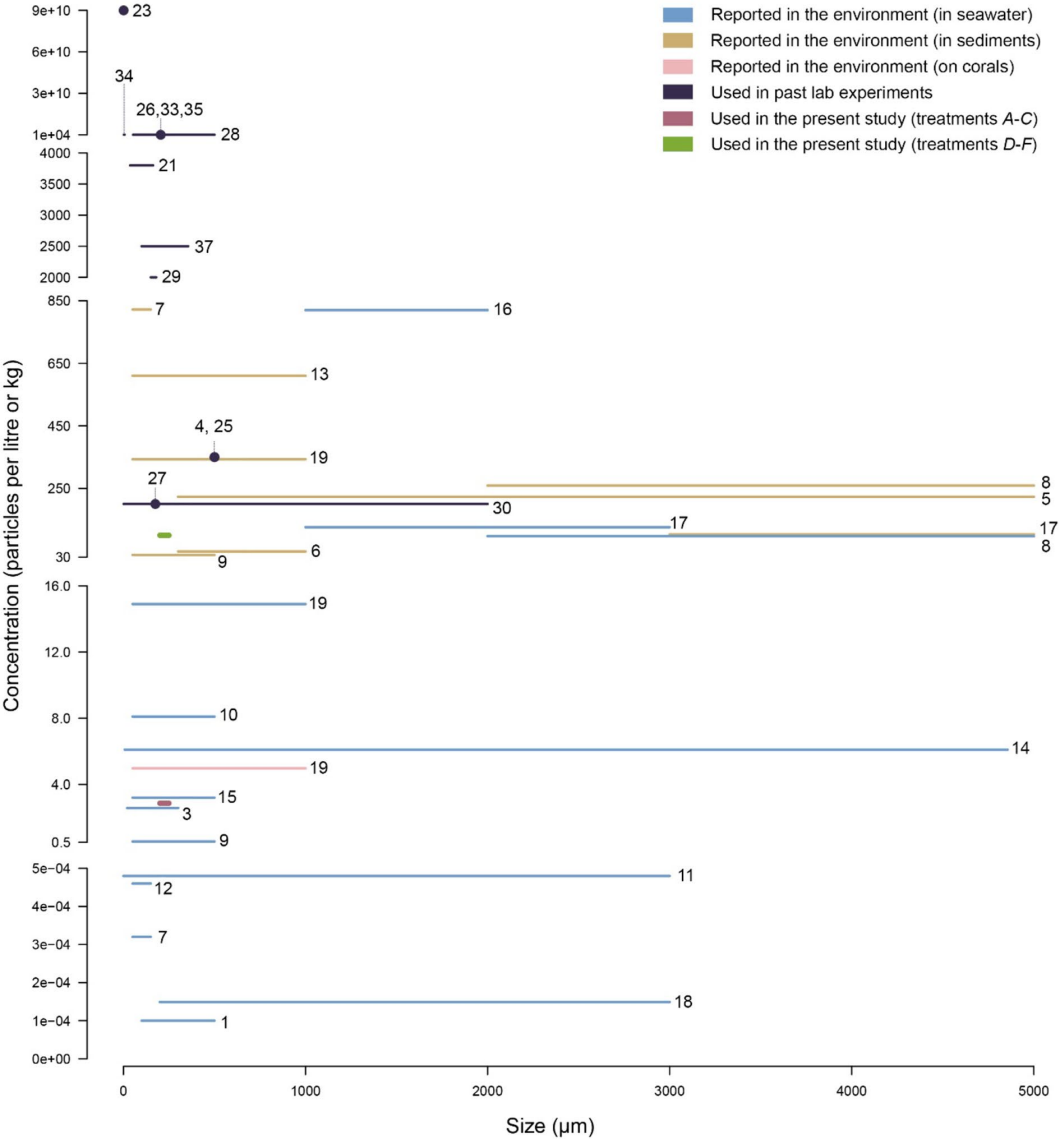
Microplastic concentrations found on reefs and used in coral laboratory experiments. Overview of all reported microplastic sizes (μm) and concentrations in seawater (particles L^−1^), sediments (particles kg^−1^), and corals (particles cm^−2^) on tropical coral reef systems (Supplementary Table S1). Lines are used when the cited authors report a size range, and dots when one mean size is given. When concentration ranges were given in published articles, we opted for plotting the maximum value for visual convenience. Conditions simulated in all experiments exposing corals to microplastics (published by October 2021, Supplementary Table S3), as well as those used in the present study (Supplementary Table S2) are presented for a rapid visual assessment of their environmental relevance. Numbering of references corresponds to the list of cited references in the supplementary material.

By comparing behavioural responses of corals to microplastics, sediments, and microzooplankton, applied in moderate amounts, our study contributes a new layer to the growing field of knowledge on biota-microplastic interactions (Fig. 5). We demonstrate that, applied in moderate amounts, PET microplastics (200–250 µm) are not as effectively sloughed away by corals as sediments but they do not affect corals’ sediment shedding rates. Further, microplastics are ingested at higher rates in the presence of natural prey than when occurring alone, yet are consistently avoided by corals in favour of natural prey, with most microplastics (93 ± 4.0%) being egested within 48 h. PET microplastics adsorb heavy metals and persistent organic pollutants from seawater^47–49^. Whether the behavioural polyp responses, and microplastic shedding/feeding rates observed here would hold in the presence of biofouled or contaminated microplastics, remains untested. Further investigation is also needed to determine whether the residence time of microplastics on coral surfaces and inside coral polyps is sufficient to desorb toxic substances and cause satiation.

*S. pistillata,* a tropical coral of relatively small polyps, reacted more adversely to microplastics than to sediments through polyp retraction, but showed no further indication of sub-lethal stress. Whether microplastics elicit more oxidative stress than sediments or have more severe effects on the photosynthesis/respiration ratio and photosynthetic yield when both are added at environmentally relevant concentrations, remains to be tested. When deposited separately from sediments, microplastics remained longer on coral surfaces than sediments. This occurred both through adhesion facilitated by the highly electrostatic behaviour of microplastics^50^ and prolonged and repeated inspections of microplastics by polyp tentacles. Instead, when deposited alone on the corals, sediments often remained uninspected on the coenosarc before being actively or passively sloughed off. It is likely that the fact that microplastics are denser than sediment particles, also contributed to their longer residence times on the corals. The relatively longer inspection times towards microplastics and relative indifference towards sediments may respond to the release of unfamiliar chemical signals by microplastics. Organisms reliant on chemosensory cues to locate and trap their prey (e.g. corals) are thought to be at a particularly high risk of ingesting microplastics colonised by biofilms with attractive chemical signals^51^. Unfouled microplastics (as used in the present study) are known to trigger more coral phagostimulants than fouled microplastics^29^. Our study suggests that unfouled irregular PET microplastics have chemical cues warranting polyp attention. We do, however, argue that these may not necessarily be phagostimulants, which would have triggered ingestion more often than observed here. Rather, these appeared to be substances signalling the unsuitability of PET particles as prey. This hints at a high capacity of corals to discriminate unfouled microplastics from food and sediments.

The branching morphology of *S. pistillata* likely aided the tendency of particles to fall off corals passively through gravity and water motion. This may explain why *S. pistillata* did not use strong microplastic rejection mechanisms (e.g. tissue or polyp inflation). Instead particles were shed by gentle tentacle movement as well as via mucus shedding and entrapment. Mucus concentrates nutrients making them available for a variety of mucus feeders including invertebrates and fish, some of which are mucus specialists^52,53^. We thus argue that coral mucus can be an important vehicle of microplastic transport to coral mucus feeders. Further, toxic chemicals tend to be loosely bound to microplastics and may thus easily desorb^54^ onto the coral mucus. Coral mucus has in fact been highlighted as likely adsorbent of aromatic hydrocarbons and heavy metals^55^. The extent to which mucus feeders discriminate or ingest contaminated mucus requires further research. Furthermore, mucus functions not only as a physicochemical barrier but also as a medium for bacteria and potential pathogens^55^. Whether coral mucus facilitates the transfer of pathogenic microbes from microplastics to corals and mucus feeders deserves attention.

We hypothesised that in corals exposed to microplastic-contaminated sediments, mechanisms for sediment rejection would be delayed or engaged in microplastic inspection and/or rejection. Contrary to this expectation, and although microplastics engaged chemoreception mechanisms, they did not retard sediment cleansing. This is encouraging, given that sedimentation can smother coral tissues, inhibit photosynthesis by endosymbionts and reduce feeding^26,56,57^. Conversely, sediments appeared to speed up microplastic elimination. It would therefore seem plausible that sediments can either affect the electrostatic nature of microplastics, or mask the chemical signals of microplastics thus avoiding repeated and prolonged tentacle inspection. The first option seems unlikely considering that electrostatic metal/plastic separators are routinely used to separate microplastics and sediments based on their electrostatic and conductive properties, respectively. However, complete separation requires dry sediments^58^, which implies that seawater may affect the conductivity/electrostatic behaviour of sediment and microplastics particles when mixed. The possibility of sediments masking the chemical signature of microplastics seems less likely, given that, when mixed with sediments, microplastics touched just as many polyps as when alone (Fig. 3f).

While microplastics did not affect the sediment clearing function of healthy small-polyped branching corals, this observation may not be directly generalizable across coral species, growth forms, life stages, and unhealthy or wounded corals. Further work should corroborate whether sediment cleansing is also undisturbed by microplastics for more encrusting coral forms, or forms with further convexities at the colony or microarchitecture scale. Sediment clearing is, for instance, more challenging for bleached than for healthy corals^59^. Moreover, under heat stress *S. pistillata* loses mucocytes in the epithelium and therefore the ability to reject particles via mucociliar transport^60^. The role of mucus in removing sediments and/or microplastics from corals may not remain constant in a warming ocean and this deserves attention. Interferences with the roles of mucus in mucociliary feeding or sunscreen protection are specially concerning for thermally-bleached corals^55^. Further, our conclusions regarding the effect of microplastics on sediment shedding efficiency do not warrant extrapolation to situations in which corals are exposed to repeated influxes of nutrient-rich microplastic-contaminated sediments in reefs with low water motion. Corals produce mucus in response to sedimentation stress^55^ and (as shown here) as a microplastic-shedding mechanism. Mucus production is, however, energetically very costly^57^. There may be microplastic pollution or sedimentation rate thresholds beyond which sediment and microplastic particles hinder coral rejection mechanisms, and/or compound each other’s impact on corals. Exposure to nutrient-rich sediment, for instance, can lead to rapid degradation of coral mucus and microbially-mediated mortality^61^. Microplastics act as rafts of microbial colonisation and may have significant impacts on environmental microbiomes^62^.

The experiments of this study clearly indicate that heterotrophy rates were three times higher than microplastic ingestion rates when either particle type occurred in isolation. Because of the different particle concentrations used, comparing heterotrophy rates between corals exposed to microzooplankton and corals exposed to *A. salina* cyst-microplastic combinations is biased. Thus, differences in particle concentrations between treatments need to be considered when comparing heterotrophy rates in corals exposed to cysts alone (at 100 items L^−1^) and corals exposed to cysts (at 50 items L^−1^) combined with microplastics. When cysts and microplastics co-occurred at half the concentration each (50 items L^−1^), heterotrophy rates were halved, whereas microplastic ingestion rates increased threefold (Supplementary Table S4). However, exposed to 50:50 cysts:microplastic mixtures and to uneven mixtures dominated by microplastics (at 75 microplastic particles L^−1^) corals fed selectively on the cysts and avoided microplastics. Hence, our results contradict previous accounts of the temperate coral *A. poculata* preferentially ingesting microbeads offered at 17,000 microbeads L^-1^ and concurrently with *A. salina* eggs, and contradict records of microplastic ingestion rates being independent of the presence of natural prey for the tropical mushroom coral *D. scruposa*^41^. The marked preference of *S. pistillata* for natural prey over plastic may indicate that although corals may be generalists nonselective microplastic feeders^63^, they can indeed discriminate between inedible plastics and edible prey. Similar to *S. pistillata* in colony morphology but with larger polyps, *P. damicornis* also ingested *A. salina* preferentially over microplastics when the latter was added at 2000 particles L^−132^. The egestion of ingested microplastics by corals has been repeatedly documented. Here, we also document the egestion of 93% of ingested microplastics. Importantly, starved polyps reportedly ingest indigestible items (e.g., chalk, filter paper) that well-fed polyps refuse^64,65^. Prior to our experiments, *S. pistillata* colonies were kept in a large holding tank and routinely fed with *A. salina* but also had access to dissolved nutrients and particulate matter. Experiments were thus carefully timed so that corals were not starved or overfed.

Many coral reefs worldwide exist under relatively high concentrations of suspended particulate matter, and corals living on turbid reefs can have up to 20 times greater capacity to feed on suspended particles, than conspecifics on less turbid reefs^66^. Turbid reefs are often a consequence of terrestrial runoff which may also carry copious amounts of microplastics^5^. Corals on turbid reefs might therefore be especially vulnerable to microplastic ingestion, not only because of the high pollution levels but also because they are habituated to feed profusely on particulate matter. The abundance of suspended sediments and edible particulates within turbid reefs might capture the attention of corals and thus ameliorate the risk of microplastic ingestion or adhesion. This will likely depend on local microplastic concentrations and remains to be tested. The role and potency of phagostimulants associated with microplastics in this context deserve particular attention.

The conditions in our experiment may resemble pulse depositions of one of the densest microplastic types (PET = 1.3–1.4 g cm^−3^) from the sea surface to the bottom, and/or the resuspension and subsequent redeposition of microplastics contained within reef sediments. In fact, reef sediments are typically resuspended and redeposited within a day, and this mobilisation may return contaminants to the water column^20,67^. The maximum microplastic concentration we used in treatments *A*–*C* is close to values reported in seawater in the South China Sea and the North Yellow Sea^68–70^ and several orders of magnitude higher than those known for the Great Barrier Reef and Maldives^28,71,72^ (Fig. 5). Our conclusions may thus be more informative of reef systems adjacent to large urbanised areas (e.g., Pyonyang ~ 2.9 million people) within semi-closed highly polluted seas, than of relatively “pristine” ecosystems. However, coral encounters with microplastics will ultimately be determined not only by local microplastic concentrations, but also by water flow and particle density. In terms of microplastic size, and given the general purpose of our experiments (i.e. to compare corals’ responses to microplastics and natural particles), we used a narrow yet common fraction (200–250 μm) of the full size range encountered in coral reefs (i.e. 7–5000 μm) (Fig. 5). We focused on PET particles because PET is one of the five most commonly mass‐produced polymers worldwide^2^. Primarily used to manufacture single‐use plastic bottles, it photodegrades into microplastics that readily deposit on the seabed^73,74^. While most microplastic particles begin to sink when their density reaches seawater density^75^, PET is slightly denser than seawater and thus more likely to encounter tropical corals compared to others. Together with cellophane, PET was indeed the dominant type of microplastic encountered inside corals along Eastern Hainan^76^.

This study used relatively low microplastic concentrations compared to others (Fig. 5) but focused on irregular PET particles within 200–250 µm. Further experiments are required to investigate the impact of realistic concentrations of mixtures of different polymers and size particles encountered in nature. We also incubated microplastics in artificial seawater for only 24 h prior to the experiment, which is insufficient to allow colonisation by a bacterial biofilm^77^. Because our study exposed corals to particle treatments for no longer than 24 h, all the effects observed here need to be considered short-term. Potential long-term effects cannot be judged from our experimental setup. Further, coral fragment morphology was not considered in the analysis of particle rejection efficiency. Instead we used solely a time-based metric. Whether differences in colony morphology affected particle shedding efficiency remains untested, yet is unlikely, given that all fragments were carefully cut to ensure similar size and number of branches.

Future studies should investigate the impact of chronic exposure to levels of suspended particles usually found on inshore, offshore, and marginal reefs. They should also perform a full physiological assessment of corals and consider comprehensive sets of sublethal stress metrics, and quantify the long-term impacts on heterotrophy or autotrophy/symbiosis and the coral microbiome. This will be central to confirming whether microplastics interfere with functions that are key for hard corals to persist on Anthropocene reefs. The impact of microplastics should also be contextualised relative to the impact of other pervasive toxic pollutants and the responses of various coral species and morphologies. This knowledge will be foundational to adequately dimension the threat posed by microplastics in tropical marine ecosystems, and prioritise mitigation strategies against plastic debris and other potential planetary boundaries.

## Methods

All the experimental work in this study was conducted at the Marine Experimental Ecology facility (MAREE) of the Leibniz Centre for Tropical Marine Research (ZMT).

### Study species

*S. pistillata* (Esper 1797) is a reef-building coral widely distributed across shallow (≤ 15 m) tropical Indo-Pacific reefs^78^. *S. pistillata* is a mixotroph able to fix inorganic carbon through photosynthesis by endosymbiotic zooxanthellae, and to gain nitrogen from predation on plankton and uptake of dissolved organic and inorganic nutrients^79^. In fact, *S. pistillata* depends increasingly on carbon from heterotrophic food sources with increasing depth^80^. Colonies are branching in morphology with densely and irregularly distributed small corallites (Ø = 1.0–2.0 mm)^78^. Healthy fragments of adult *S. pistillata* used for this study (n = 53) were sourced from the Marine Experimental Ecology (MAREE) aquarium facility of the Leibniz Centre for Tropical Marine Research and originally reproduced and reared in captivity (Hagenbeck Zoo, Germany).

### Origin and preparation of microplastics and sediments

Irregularly shaped polyethylene terephthalate (PET) microplastics were purchased from Goodfellow GmbH Germany (PET powder ES306030). PET density ranged from 1.3 to 1.4 g cm^−3^. PET is a common polymer used for packaging food and liquids, as well as for clothing. Due to their high density, PET particles sink faster and are more likely to interact with benthic organisms, than floating plastic polymers. Despite the fact that most microplastics in the marine environment have a biofilm^77^, we opted for using unfouled particles in this study to investigate the effects of just the plastic. PET particles ranged from 50 to 250 μm in diameter. To separate particles between 200 and 250 μm, the powder was sieved for 1 min through 250- and 200-μm sieves using a vibratory sieve shaker (RETSCH AS 200; 1.04 magnitude mm^−1^ g^−1^). This size was chosen, as it is in the natural range of prey organisms and similar to the size of the *A. salina* cysts used in the feeding experiment. To view, track, and count the microplastics with microscopes and video-cameras, we stained them in two steps using solutions of lipophilic fluorescent dye Nile Red in methanol (10 μg ml^−1^, 48 h) and acetone (1 μg ml^−1^, 48 h)^81^. A bright and even tone was achieved by staining 1 g of microplastics at a time.

Reefal sediments were obtained from calcium carbonate reef sand (Red Sea Premium Sand from Mrutzek Meeres-Aquaristik GmbH, Germany). Sediments were slightly smoother, rounder, and denser (2.7–2.9 g cm^−3^) than microplastics but within the same size range (sediments: 256.9 ± 24.3 μm microplastics: 261.35 ± 27.5 μm, Supplementary Fig. S1c-d). Both, microplastics and sediments were incubated in artificial seawater 24 h prior to any experiment to eliminate surface tension and ensure sinking.

### Coral fragmentation and husbandry

A total of 53 coral fragments of ~ 5 × 5 × 5 cm of *S. pistillata* were obtained from five parent colonies and immediately glued (EcoTech elements Coral Glue) onto ceramic coral plugs (Thrive Aquatics). All fragments were then held in a 3000 L maintenance tank at constant temperature (25 °C) and ambient light regime (12 h light and 12 h darkness) for three months prior to experiments, to allow for sufficient recovery time. Temperature and salinity were checked daily, while other water parameters were monitored once per week and adjusted when necessary (Supplementary Table S5). As the maintenance aquaria contained fish, invertebrates, and algae as well as a sandy bottom, all fragments had access to bacteria, microzooplankton, and dissolved nutrients, which are necessary for heterotrophic feeding and calcification. In addition, coral fragments were fed with *A. salina* cysts and nauplii twice per week.

### Experimental exposure of corals to microplastics and sediments

For experiments involving microplastics and/or sediments, corals were sequentially transferred from the maintenance tank into a separate 30 L glass tank subdivided by glass plates into three 10 L chambers (hereafter referred to as the experimental tank). Each coral fragment (one at a time) was held for 24 h in 8 L of filtered seawater inside the middle chamber of the experimental tank, whereas the outer chambers held freshwater, a heater, and a temperature control unit (SCHEGO Regler TRD, 220–240 V/50 Hz/Sensor 2.0) to maintain a constant temperature. A magnetic stirring plate (Thermo Scientific) was placed under the experimental tank and set at 300 rpm, and a magnetic stir bar (3.0 × 0.5 cm) was placed inside the middle chamber to ensure constant water flow. Compressed air was supplied continuously through a glass pipette at 1 bubble second^−1^. A lamp (Hydra FiftyTwo HD) positioned above the tank provided light adjusted to 15% UV, 15% violet, 75% royal blue, 55% blue, 0% green, 10% deep red and 25% cool white (i.e. 50 Watt) to optimise the fluorescence of microplastics.

Corals were acclimated to the experimental tank until full polyp expansion (i.e. ~ 2 h). Following acclimation, particles were applied once directly onto the coral surface with 1 ml syringes (Injekt F). A GoPro video camera (Hero 4) mounted on a tripod outside and in front of the experimental tank recorded still images every two seconds for 12 h. To capture polyp behaviour and ensure particle detection, the camera was used inside a GoPro underwater housing fitted with a Backscatter FLIP6 mounting, a 55 m macro lens (15 Macromate mini), a 55 mm Close-up + 10 filter, and a yellow filter. An underwater GoPro charger (PolarPro Power Grip GoPro, 6700 mAh) allowed for extended battery life and continuous use of the time-lapse mode for 12 h. The batch file renaming software Advanced Renamer (version 3.84) was used to rename the photos to the exact points in time (hh.mm.ss). Cameras captured the lateral view of the entire coral fragments. Although syringes were loaded with an identical number of particles per trial, ensuring an identical number of particles landed on all corals was practically impossible. Therefore, the number of particles visible to the camera at the onset of the experiment (hour 0) ranged between 13 and 34 across fragments, but did not differ significantly among treatments (Supplementary Fig. S5, Table S6). A total of 19 *S. pistillata* fragments were allocated to three treatments (*A*–*C*) simulating pulse exposures to microplastics reaching up to 2.86 particles L^−1^ (Supplementary Table S2). Treatments were compared to one another and to procedural controls (n = 6 fragments) to address the research questions (Fig. 1).

#### Comparing coral responses to sediments and microplastics

To determine whether sediment and microplastic particles elicit similar responses in corals, *S. pistillata* fragments were sequentially allocated to one of two treatments (*A* and *B*) with seven replicate fragments per treatment. Treatments *A* and *B* consisted of single 12-h exposures to reef sediments and stained microplastics, respectively (Fig. 1). To account for likely effects of the experimental setup, six additional coral fragments were exposed to control conditions (i.e. transferred from the maintenance to the experimental tank but without adding particles). All corals were photographed with a stationary GoPro video camera every hour. Photos were scored to measure the proportion of fully retracted polyps as a colony-scale behavioural response to stress. Polyp retraction is a protective behavioural response of corals against predation, desiccation, or irradiance^82,83^ and an important change in coral condition and activity level that may precede partial tissue loss and/or complete mortality following exposure to hydrocarbons^84^. Explicit metrics of coral stress (e.g., photosynthesis/respiration ratios^27^) or signs of the activation of defence mechanisms against stress or stimuli (e.g. levels of reactive oxygen species^85^) were outside the scope of the present study. The total numbers of polyps inside the camera’s field of view were counted once per colony. The limited availability of *S. pistillata* fragments at the time of the experiment precluded our capacity to test whether the coral reacts differently to stained and unstained microplastics. However, a parallel experiment on *Pocillopora damicornis* confirmed no significant differences in the proportion of retracted polyps over time between fragments exposed to stained and unstained irregular PET microplastics (Supplementary Fig. S6, Table S7).

#### Testing whether microplastics retard corals’ efficiency to shed reefal sediments

To determine whether microplastics retard the efficiency of corals to shed reef sediments off their surface, we allocated five additional *S. pistillata* fragments to treatment *C* which consisted of a single 24-h exposure to a 50:50 mixture of reef sediments and microplastics. Corals under treatment *C* were then compared to those under treatment *A* (sediments) (Fig. 1). For this comparison, the time lapse photographs were scored using ImageJ (version 2.0.0)^86^. The trajectory of each sediment particle from first contact with the coral to full detachment was tracked on each time-lapse sequence, using the ImageJ plug-in MTrackJ (version 1.5.1)^87^. The last hour at which each particle was sighted on the coral colony was noted.

### Experimental exposure of corals to microplastics and food

The brine shrimp *Artemia* spp. is a digestible and frequently used food source for aquarium corals^88^. Experiments exposing corals to microplastics and/or decapsulated *A. salina* cysts (purchased at Interaquaristik, Germany) were performed sequentially over 24 h in the dark, in sets of four fragments at a time, placing each fragment inside 1-l Weck jars filled with filtered seawater (Supplementary Fig. S1a). For flow and aeration, the jars containing small stirring bars were placed on a magnetic stirring plate (300 rpm) and continuously supplied with compressed air through glass pipettes (1 bubble second^−1^). The setup was immersed in a freshwater bath held at a constant 25.0 (± 0.03) °C with aid of a heater/temperature control system and a flow pump (Aquabee UP500) set at 500 l h^−1^. To avoid disrupting water flow within the jars or overcrowding them with surfaces where particles could become entrapped, coral fragments were suspended within the centre of the jars using 0.5 mm nylon thread (Supplementary Fig. S1a). Following the attachment of the nylon threads, the experiments commenced once the polyps were fully distended (~ 1 h). All jars contained the exact same volume of water (1 L) and were sealed with glass lids for the duration of the experiment to avoid external contamination and water evaporation. A total of 28 *S. pistillata* fragments were allocated to treatments *D*–*G* (n = 7 per treatment, Fig. 1), adding particles (microplastics and/or *A. salina* cysts) with 1-ml syringes. In all treatments, particles were counted by hand through a microscope and corals were exposed to a total of 100 particles, thus achieving total concentrations of up to 100 particles L^−1^ (Supplementary Table S2).

To assess the feeding rates of the coral fragments, the total number of microplastic particles and *A. salina* cysts remaining in the water after 24 h was counted. To avoid particle loss, nylon threads, stir bars, glass pipettes, and coral fragments were thoroughly rinsed with filtered seawater into a larger beaker. The contents of this beaker were then filtered through cellulose filters (Whatman 597) using glass funnels. Weck glasses and beakers were then rinsed with deionised water into the funnels three times to ensure the transfer of all particles. The particles on the filters were counted manually under a stereomicroscope (Zeiss SteREO Discovery.V8) with an additional cold-light source (Zeiss CL 4500 LED CRI90). All fragments that had consumed microplastics in 24 h were placed back into fresh Weck jars and monitored for an additional 24 h. Egested microplastics were recovered repeating the filtration procedure and counted.

Feeding rates (i.e. number of particles consumed) were normalised by the corals’ surface area quantified from three-dimensional fragment models. To this aim, fragments were photographed from all sides and with a size reference, capturing 30–40 highly overlapping images per fragment (Supplementary Fig. S1b). Models were reconstructed using Autodesk ReCap Pro/Autodesk ReCap Photo version 5.0.40.

#### Testing the effect of microplastics on corals’ natural feeding rate

To test whether coral heterotrophy and microplastic ingestion rates differ and whether the occurrence of microplastics within microzooplankton interferes with heterotrophy, we applied a two-pronged approach. First, we compared the overall and per-particle feeding rates of corals (items ingested cm^−2^ 24 h^−1^) among fragments presented with *A. salina* cysts alone (treatment *D*), microplastics alone (treatment *E*), and 50:50 mixtures of cysts and microplastics (treatment *F*) (Fig. 1). A total of 100 items were added in all cases, amounting to 100 cysts L^−1^ in treatment *D*, 100 microplastic particles L^−1^ in treatment *E*, and 50 cysts L^−1^ and 50 microplastic particles L^−1^ in treatment *F*. Second, we computed Manly’s α selectivity indices per particle type and compared them between fragments exposed to even mixtures of cysts and microplastics (treatment *F*), and fragments exposed to 25 cysts L^−1^ and 75 microplastic particles L^−1^ (treatment *G*). Microplastics (261.35 ± 27.5 μm) and *A. salina* cysts (252.08 ± 21.3 μm) were within similar size ranges (Supplementary Fig. S1e-f).

### Statistics and reproducibility

All statistical analyses were conducted in R (version 3.6.2)^89^.

To test whether corals show similar levels of visible stress (determined by the proportion of retracted polyps) when exposed to sediments and microplastics, a generalised additive mixed-effects model (GAMM) with a binomial distribution was fitted using the R package *gamm4*^90^. A smoother for *time* (*hours*) and the three-level factor *treatment* (i.e. control, *A*—sediments, and *B*—microplastics) allowing for their interaction were included as fixed predictors. Two random effects were included to account for (1) the dependency among repeated measures made on each of the coral fragments and (2) the variation added by the percent of particles remaining at each hour relative to total number added at the start of the experiment (*hour* = 0). The Akaike Information Criteria (AIC) confirmed eight as the optimal number of knots and that the model with a smoother per *treatment* was superior to a model with a single smoother. Model residuals were plotted against fitted values to confirm homoscedasticity and against all variables in the model to verify independence (Supplementary Fig. S7)^91^. Fitting a binomial GAMM instead of a binomial GLMM was justified, as residuals from the latter were significantly nonlinearly related to time (*hours*) (F = 3.028, p = 0.003)^92^.

To test whether corals (1) need more time to fully shed off microplastics than sediments, (2) take longer to rid themselves of sediments contaminated with microplastics than of uncontaminated sediments, and (3) take a similar amount of time to reject microplastics when they occur alone or combined with sediments, we applied three separate survival analyses fitting Kaplan–Meier (KM) curves^93^. To this end, we used the *survfit* function of the *survival* R package^94^ . The first analysis considered the hourly number of particles remaining on corals subjected to sediments (treatment *A*) and microplastics (treatment *B*). The second analysis considered the hourly counts of remaining sediment particles on corals exposed to sediments (treatment *A*) and sediments with microplastics (treatment *C*). Lastly, the third analysis considered the hourly counts of remaining microplastic particles on corals subjected to microplastics (treatment *B*) and sediments with microplastics (treatment *C*). In all three cases, a KM curve was fitted per treatment, and survival time was measured as hours from particle addition. The survival time curves were compared using a random-effects Cox proportional hazard model using the *nested.coxph* function in the *NestedCohort* R package^95^. Here, *treatment* was fitted as a fixed effect and *fragment* included as a random effect to account for the dependence among particle survival times observed within each coral fragment. The significance of the factor *treatment* was assessed through a Wald test. To explore whether the probability of particle residence was linked to the number of polyps touched per particle, we also compared the number of polyps touched by sediments and microplastics among treatments *A*–*C*. Initial Poisson generalised linear models (GLMs), including *treatment* and *coral surface area* as predictors were underdispersed (i.e. dispersion parameters = 0.26–0.41), most likely due to the dominance of 1 s. We therefore fitted hurdle models with a Poisson error distribution and log-link function, suitable for underdispersed one-inflated data in a Bayesian framework using the R package *brms*^96^. Models considered *treatment* and *coral surface* area as predictors. The posterior distributions of the model parameters were estimated using Markov chain Monte Carlo (MCMC) methods by constructing four chains of 4000 steps with a warm-up of 1000 steps. For all models we inspected the MCMC chains for convergence and model fit. We used the default, noninformative priors set by the *brms* function.

To test whether (1) coral heterotrophy rates were higher than coral microplastic ingestion rates (when microzooplankton and microplastics occur in isolation at 100 items L^−1^), and (2) both heterotrophy and microplastic ingestion rates change when microzooplankton and microplastics are evenly mixed at 50 items L^−1^ each, three generalised least-squares models (GLS) were fitted. All of these included a variance structure of the form *varIdent* to account for heterogeneity of variance among treatments^97^.

To determine whether corals prefer microzooplankton over microplastics when these are mixed in even or uneven proportions, we computed Manly’s α selectivity per prey type and treatment for treatments *F* (50:50 *A. salina*:microplastic mixtures) and *G* (25:75 *A. salina*: microplastic mixtures) accounting for food source depletion^98^ using the R package *selectapref*^99^. Prior verification of normality and homoscedasticity, we then ran one-sample T-tests to determine whether Manly’s α per prey type and treatment were significantly different from the value expected for no selectivity (i.e. 0.50). Furthermore, as variances differed between treatments *F* and *G*, we conducted a Welch’s two-sample t-test to determine whether the strength of Manly’s α selectivity for *A. salina* differed between these treatments.

## Supplementary Information

Supplementary Information.

## Data Availability

The datasets generated and/or analysed during this study as well as the R codes are available in Dryad through the 10.5061/dryad.ngf1vhht8.
