# Supplementary material for: No short-term effect of sinking microplastics on heterotrophy or sediment clearing in the tropical coral Stylophora pistillata
Source: Sci Rep. 2022 Jan 27;12:1468. doi: 10.1038/s41598-022-05420-7 (PMC8795188; doi:10.1038/s41598-022-05420-7)
Supplement: Supplementary file 1 — Supplementary Information. [file 41598_2022_5420_MOESM1_ESM.pdf]

No short-term effect of sinking microplastics on heterotrophy or sediment clearing in the tropical coral *Stylophora pistillata*

Sonia Bejarano, Valeska Diemel, Anna Feuring, Mattia Ghilardi, Tilmann Harder

Supplementary material

Contents in order of appearance (in main text)

**Table S1**	Microplastic concentrations reported in the environment on coral reef areas since 2015 up to February 2021.
**Table S2**	Mean (±SE) single-pulse concentrations of microplastics, sediments, and *Artemia salina* cysts reached per treatment at the start of the experiments.
**Table S3**	Microplastic concentrations used in previously published experiments exposing Corals to microplastics, compared to those used here.
**Figure S1**	Photographs of the a) experimental setup of the feeding experiments, b) corals' 3D models, c) calcareous sediment particles, d) stained PET microplastics, e) decapsulated *A. salina* cysts, g) Microplastic particles trapped in mucus strains and in h) gas bubbles.
**Figure S2**	Output of Bayesian Poisson hurdle model for numbers of polyps touched by microplastics and sediments when these are added separately to corals.
**Figure S3**	Output of Bayesian Poisson hurdle model for numbers of polyps touched by sediments when these are added separately to corals or in 50:50 mixtures with microplastics.
**Figure S4**	Output of Bayesian Poisson hurdle model for numbers of polyps touched by microplastic particles when these are added separately to corals or in 50:50 mixtures with sediments.
**Table S4**	Output of models comparing i) heterotrophy rates in corals exposed to *A. salina* alone at 100 items L^{-1} and corals exposed to even mixtures *of A. salina* and microplastics at 50 items L^{-1} each and ii) microplastic ingestion rates in corals exposed to only microplastics at 100 items L^{-1} and corals exposed to even mixtures *of A. salina* and microplastics at 50 items L^{-1} each.
**Table S5**	Mean values (± SE) of selected physicochemical parameters of the seawater inthe coral maintenance tank for the duration of the experiment 01.08. - 31.12.2018
**Figure S5**	Mean number of particles (± SE) visible to the cameras at the beginning of the experiments (hour = 0) exposing *S. pistillata* fragments to reef sediments (treatment *A*), microplastics (treatment *B*), and 50:50 mixtures of reef sediments and microplastics (treatment *C*). A Poisson GLM detected no significant differences among treatments (p = 0.39).
**Table S6**	Outputs of the linear model testing whether the number of particles cm^{-2} coral differed significantly among treatments *A* - *C*.
**Figure S6**	Proportion of retracted polyps recorded hourly for 12 hours in a parallel experiment run on *Pocillopora damicornis* exposed to a) stained and b) unstained irregular PET microplastics. No significant differences were observed among hours or between the type of particles (Table S7).
**Table S7**	Statistical outputs of a binomial generalised linear model (GLMER) fitted to data obtained during a parallel experiment exposing *P. damicornis* to *stained* (n = 9 fragments) and *unstained* (n = 5 fragments) irregular PET microplastics. The model tests whether the proportion of retracted polyps within colonies changed hourly for 12 hours, and whether this change was contingent on microplastic staining.
**Figure S7**	Model validation plots for the GAMM with binomial distribution fitted to test whether the proportion of polyps retracted over time differed when exposed to control experimental conditions, sediments, and microplastics.
**References cited**	

Table S1

Table S1. Microplastic concentrations reported in the environment on coral reef areas since 2015 up to February 2021.

Concentration (Location)	Reference
0.0001 particles L^{-1} seawater (GBR)	Hall et al. 2015[1]
0.74 particles m^{-2} sea surface area	Connors 2017[2]
2.57 particles L^{-1} seawater (South China Sea)	Cai et al. 2018[3]
0 to 2.28 mg L^{-1} seawater (Mediterranean)	Chapron et al. 2018[4]
171.7 ± 57.6 to 223 ± 51.4 particles kg^{-1} in sediments (Hong Kong)	Cheang et al. 2018[5]
48.3±13.98 particles kg^{-1} sediments (Indonesia)	Cordova et al. 2018[6]
0.0032 particles L^{-1} seawater, 822 articles kg^{-1} in beach sand (Maldives)	Saliu et al. 2018[7]
259 particles kg^{-1} sediments, 97 particles L^{-1} seawater (Southeast India)	Jeyasanta et al. 2020[8]
0.55 particles L^{-1} seawater (North Yellow Sea)	Zhu et al. 2018[9]
8.1 particles L^{-1} seawater (South China Sea)	Huang et al. 2019[10]
0.00048 particles L^{-1} (GBR)	Jensen et al. 2019[11]
0.00012 - 0.00046 particles L^{-1} seawater (Maldives)	Saliu et al. 2019[12]
40 – 610 particles kg^{-1} reef sand (South China Sea)	Zhang et al. 2019[13]
6.1 particles L^{-1} seawater (South China Sea)	Ding et al. 2019[14]
1.25 – 3.2 particles L^{-1} seawater (South China Sea)	Nie et al. 2019[15]
4.5 particles L^{-1} seawater (Maowei Sea)	
820 particles kg^{-1} sediment (Philippines)	Bucol et al. 2020[16]
60-127 particles L^{-1} seawater, 50 to 104 particles kg^{-1} in sediments (Southwest India)	Patterson et al. 2020[17]
8.1 particles L^{-1} seawater (South China Sea)	Tan et al. 2020[18]
14.9 particles L^{-1} seawater, 343 particles kg^{-1} sediments, 4.97 particles cm^{-2} coral (South China Sea)	Tang et al. 2021[19]

Table S2

Table S3. Mean (±SE) single pulse concentrations of microplastics, sediments, and *A. salina* reached per treatment at the start of the experiments in particles per litter of water in the experimental tank.

Treatment	Mean microplastic concentration particles L^{-1}	Mean sediment concentration particles L^{-1}	Mean *A. salina* concentration cysts L^{-1}
A - Sediments	0.00 (± 0.00)	2.64 (± 0.33)	-
B - Microplastics	2.86 (± 0.41)	0.00 (± 0.00)	-
C - Sediments and microplastics (50:50)	1.68 (± 0.16)	1.45 (± 0.09)	-
D - *Artemia*	0.00 (± 0.00)	-	100.00 (± 0.00)
E - Microplastics	100.00 (± 0.00)	-	0.00 (± 0.00)
F – *Artemia*:microplastics 50:50	50.00 (± 0.00)	-	50.00 (± 0.00)
G - *Artemia*:microplastics 25:75	75.00 (± 0.00)	-	25.00 (± 0.00)

Table S3

Table S2. Microplastic concentrations used in previously published experiments exposing corals to microplastics, compared to those used here.

Concentration	Reference
0.1 - 0.3 g L^{-1}	Hall et al. 2015[1]
0.3 g L^{-1}	Allen et al. 2017[20]
0.1 g L^{-1} ≈ 3799 particles L^{-1}	Reichert et al. 2017[21]
0.03 g L^{-1}	Hankins et al. 2018[22]
0.05 g L^{-1} ≈ 9 x 10^{10} particles L^{-1}	Tang et al. 2018[23]
350 particles L^{-1}	Chapron et al. 2018[4]
0.05, 0.10, and 0.15 g L^{-1}	Syakti et al. 2019[24]
0.002 g L^{-1} ≈ 350 particles L^{-1}	Mouchi et al. 2019[25]
0.2 g L^{-1} ≈ 17000 particles L^{-1}	Rotjan et al. 2019[26]
0.00025 g L^{-1} ≈ 200 particles L^{-1}	Reichert et al. 2019[27]
13645 particles L^{-1}	Martin et al. 2019[28]
2000 particles L^{-1}	Axworthy and Padilla-Gamino 2019[29]
200 particles L^{-1}	Berry et al. 2019[30]
0.38 g L^{-1}	Corona et al. 2020[31]
0.05 g L^{-1}	Jiang et al. 2020[32]
5000 - 50000 particles L^{-1}	Lanctot et al. 2020[33]
10 x 10^7 particles L^{-1}	Okubo et al. 2020[34]
0.001 to 0.01 g L^{-1} ≈ ~0.5 x 10^5 to 4 x 10^5 particles L^{-1}	Rocha et al. 2020[35]
0.0001 g L^{-1}	Mendrik et al. 2021[36]
2.5-2500 particles L^{-1}	Reichert et al. 2021[37]
Treatment A - 0.00 (± 0.00) particles L^{-1}	This study
Treatment B - 2.86 (± 0.41) particles L^{-1}	
Treatment C - 1.68 (± 0.16) particles L^{-1}	
Treatment D - 0.00 (± 0.00) particles L^{-1}	
Treatment E - 50.00 (± 0.00) particles L^{-1}	
Treatment F - 75.00 (± 0.00) particles L^{-1}	
Treatment G - 100.0 (± 0.00) particles L^{-1}	

Figure S1

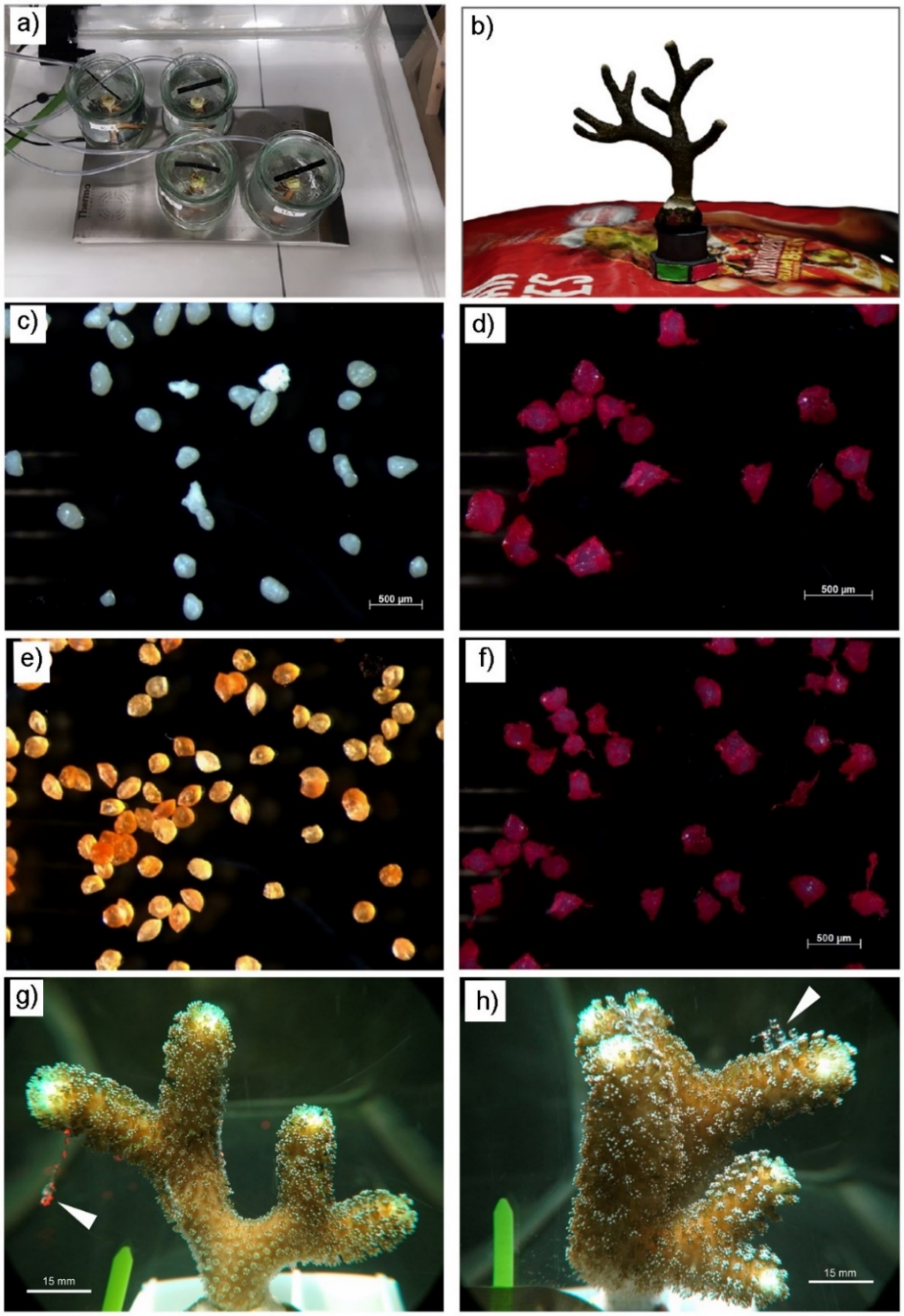

Fig. S1. a) Experimental setup of the feeding experiments including the 1L Weck jars within which fragments were suspended. b) Example of the fragments' 3D-model generated in Autodesk ReCap Pro in order to compute colony surface area. c) Reef sediment particles, d) stained PET microplastics used in the experiments exposing corals to sediments and/or microplastics, e) decapsulated *A. salina* cysts, f) stained PET microplastics used in the experiments exposing corals to *A. salina* and/or microplastics, all photographed through a stereomicroscope. g) Microplastic particles trapped in mucus strains and in h) gas bubbles. Gas bubbles were not exclusively (yet more often) produced by corals exposed to microplastics, but also occasionally produced by control corals likely in response to the lighting used in the experimental set up.

Figure S2

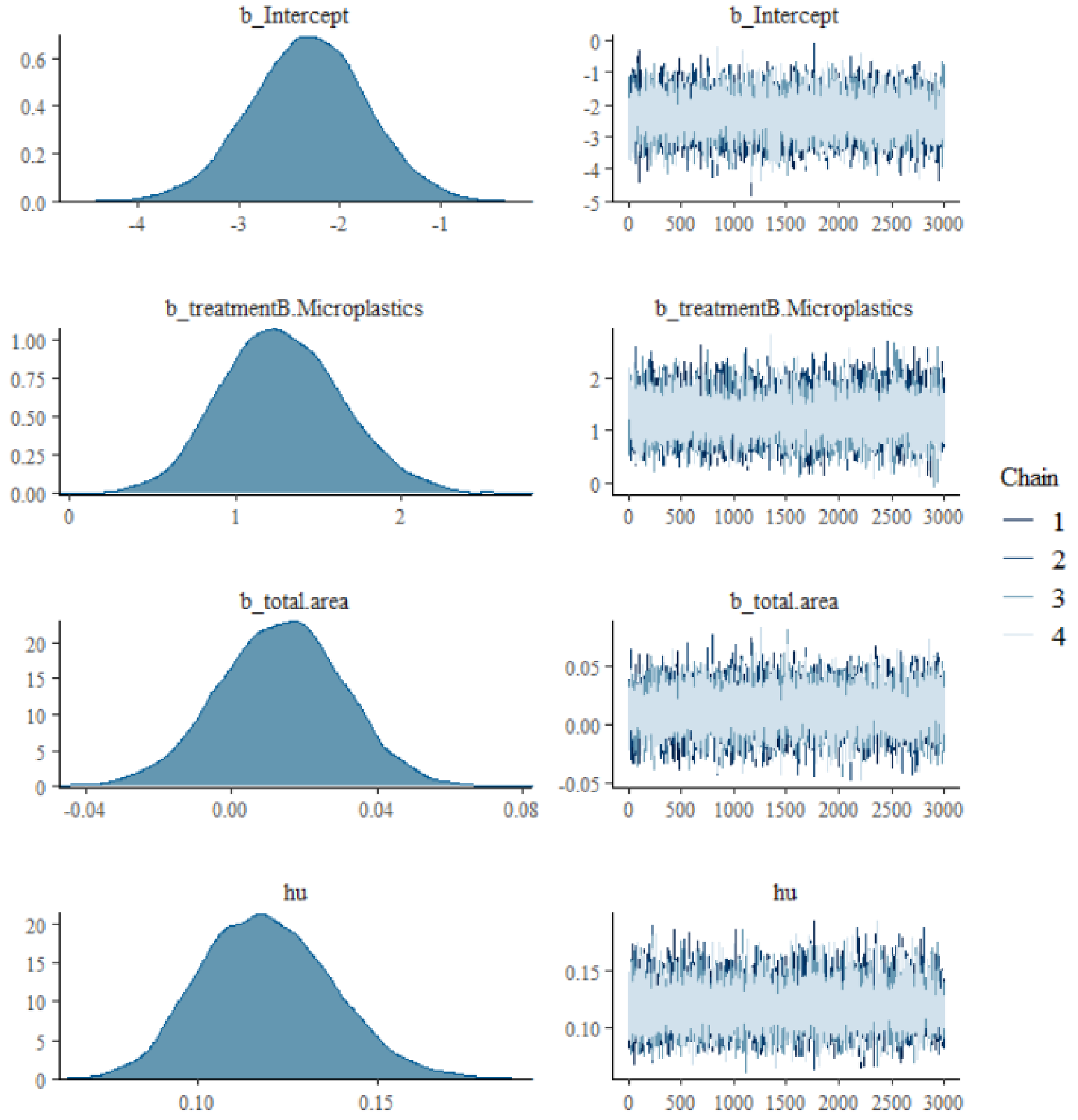

**Outputs of Bayesian hurdle model**				
Population-level effects	Estimate	SE	L95% CI	U95% CI
Intercept (*Treatment A* - Sediments)	-2.31	0.58	-3.46	-1.19
Treatment B - Microplastics	1.28	0.37	0.59	2.04
Colony surface area	0.01	0.02	-0.02	0.05
Hurdle probability (hu)	0.12	0.02	0.09	0.16

Fig. S2. Posterior distributions and degree of chain mixing per parameter in the Bayesian hurdle model testing whether, when presented separately to the corals (in treatments *A* and *B*), sediments and microplastics differ in the number of coral polyps touched per particle. Treatment *A*-Sediments is nested in the intercept, and *b_total.area* refers to the corals' surface area.

Figure S3

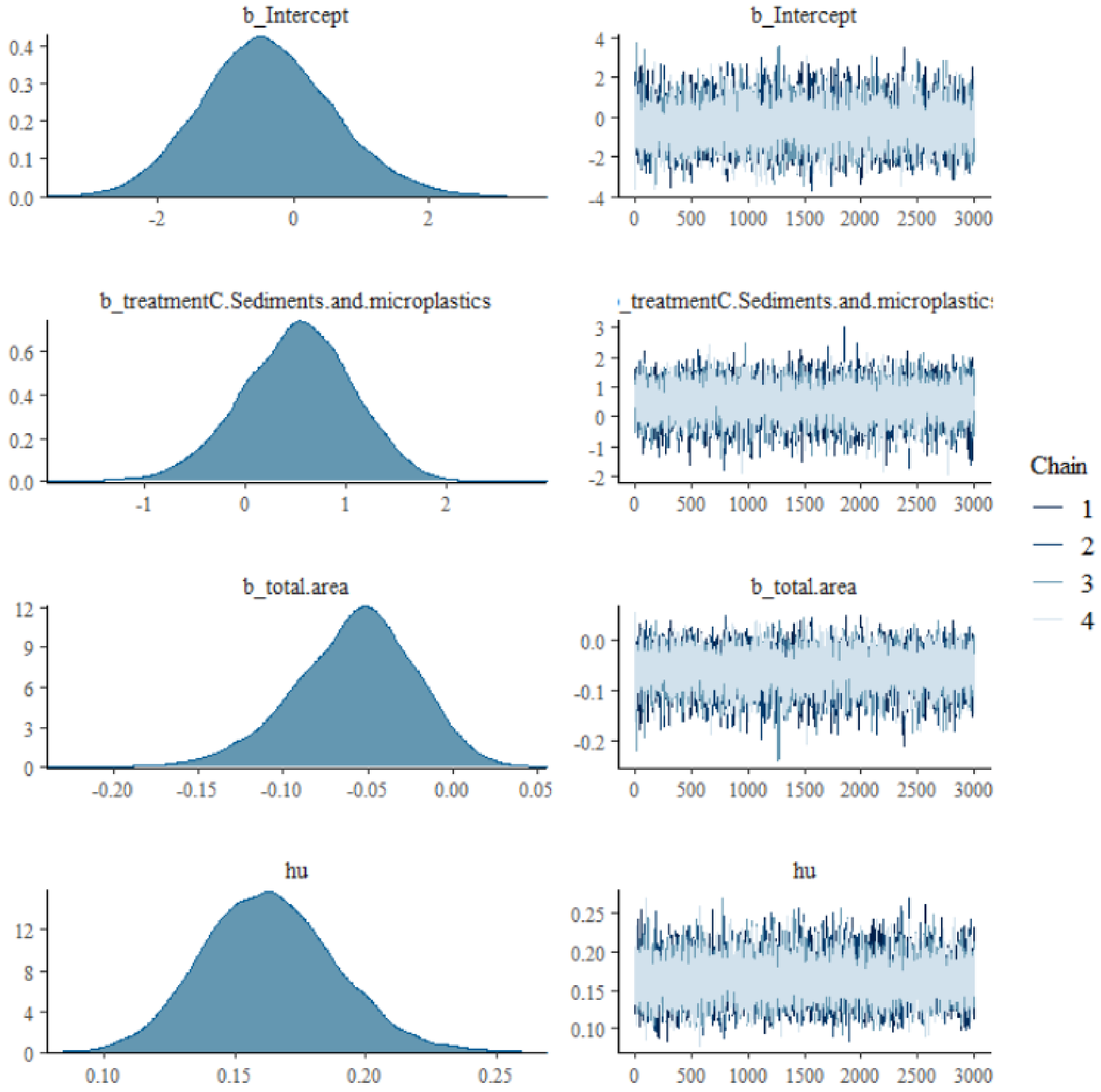

**Outputs of Bayesian hurdle model**				
Population-level effects	Estimate	SE	L95% CI	U95% CI
Intercept (*Treatment A* - Sediments)	-0.41	0.96	-2.25	1.56
Treatment C - Sediments and microplastics	0.51	0.56	-0.65	1.57
Colony surface area	-0.06	0.04	-0.13	0.01
Hurdle probability (hu)	0.16	0.03	0.12	0.22

Fig. S3. Posterior distributions and degree of chain mixing per parameter in the Bayesian hurdle model testing whether sediments touch a different number of coral polyps when added separately onto the corals (treatment *A*) or in combination with microplastics (treatment *C*). Treatment *A*-Sediments is nested in the intercept, and *b_total.area* refers to the corals' surface area.

Figure S4

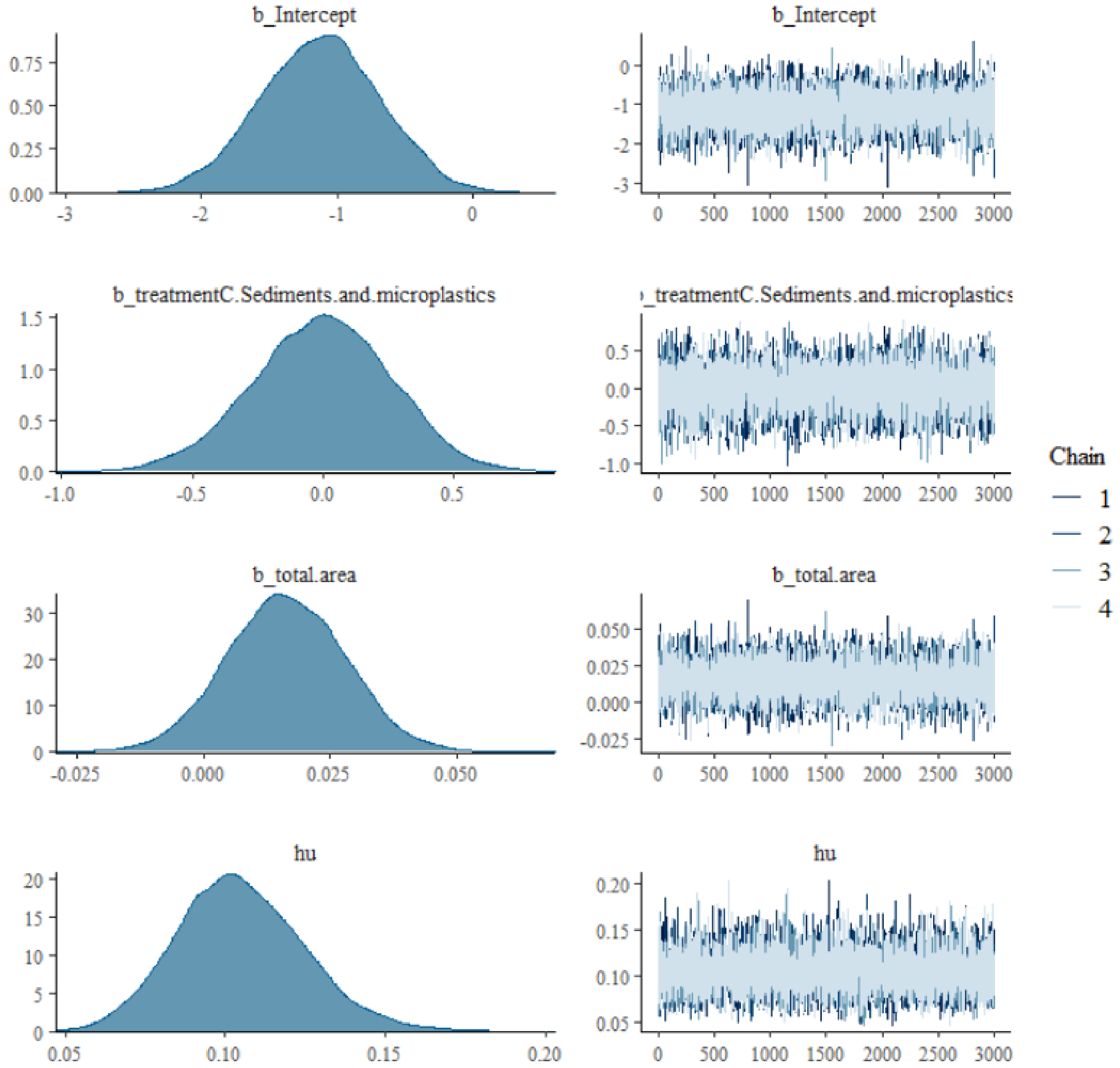

**Outputs of Bayesian hurdle model**				
Population-level effects	Estimate	SE	L95% CI	U95% CI
Intercept (*Treatment B* - Microplastics)	-1.13	0.44	-2.00	-0.29
Treatment C - Sediments and microplastics	-0.01	0.27	-0.54	0.50
Colony surface area	0.02	0.01	-0.01	0.04
Hurdle probability (hu)	0.10	0.02	-0.01	0.04

Fig. S4. Posterior distributions and degree of chain mixing per parameter in the Bayesian hurdle model testing whether microplastic particles touch a different number of coral polyps when added separately onto the corals (treatment *B*) or in combination with sediments (treatment *C*). Treatment *B* - Microplastics is nested in the intercept, and *b_total.area* refers to the corals' surface area.

Table S4

Table S4. Output of models comparing i) heterotrophy rates in corals exposed to *A. salina* alone at 100 items L^{-1} and corals exposed to even mixtures of *A. salina* and microplastics at 50 items L^{-1} each, and ii) microplastic ingestion rates in corals exposed to only microplastics at 100 items L^{-1} and corals exposed to even mixtures of *A. salina* and microplastics at 50 items L^{-1} each.

**Outputs of statistical models**				
Linear model comparing heterotrophy rates				
	Coefficient	SE	t-value	Pr (>|t|)
Intercept (treatment *E*)	0.9051	0.0296	30.595	9.33e-13
Treatment *F*	-0.3386	0.0418	-8.093	3.34e-06
GLS (Allowing for heteroscedasticity across treatments) comparing microplastic ingestion rates				
	Coefficient	SE	t-value	Pr (>|t|)
Intercept (treatment *E*)	0.2663	0.0130	20.5661	0.0000
Treatment *F*	0.7522	0.0553	13.5953	0.0000

Table S5

Table S5. Mean values (± SE) of selected physicochemical parameters of the seawater in the coral maintenance tank for the duration of the experiment 01.08. - 31.12.2018

Parameter	Mean (±SE)
Temperature [°C]	25.5 ± 0.4
Salinity [ppt]	34.7 ± 0.1
pH	7.97 ± 0.07
Nitrate - NO_3 [mg L^{-1}]	1.19 ± 0.78
Phosphate - PO_4 [mg L^{-1}]	0.07 ± 0.03
Total alkalinity [mmol L^{-1}]	2114.41 ± 275.29
Hardness - KH [°dh]	5.92 ± 0.77
Calcium - Ca [mg L^{-1}]	400.13 ± 18.25
Magnesium - Mg [mg L^{-1}]	1289.46 ± 41.43
Potassium - K [mg L^{-1}]	336.02 ± 29.20
Strontium [mg L^{-1}]	2.63 ± 0.15

Figure S5

Although we the same number of particles (n = 35) was supplied to all fragments in the sediments and/or microplastics experiments, the number of particles that actually settled on the corals and were visible to the camera at the onset of the experiment (hour 0) differed across fragments. To test for potential biases this may have caused in our results, we tested whether the number of settled particles in view differed among treatments using a Poisson generalised linear model (GLM) suitable for discrete responses. The dispersion parameter of the GLM (i.e. 2.1 indicated no overdispersion, and the output revealed no significant differences among treatments ($p = 0.39$, Fig S2).

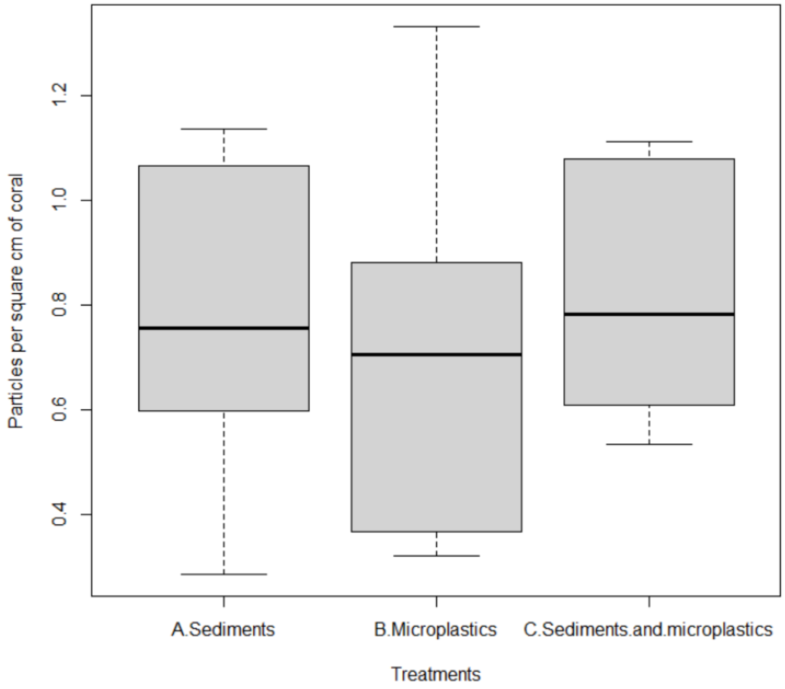

Fig S5. Mean number of particles (± SE) visible to the cameras at the beginning of the experiments (hour = 0) exposing *S. pistillata* fragments to reef sediments (treatment *A*), microplastics (treatment *B*), and a 50:50 mixture of reef sediments and microplastics (treatment *C*). A Poisson GLM detected no significant differences among treatments ($p = 0.39$).

Table S6

Table S6. Outputs of the linear model testing whether the number of particles cm^{-2} coral differed significantly among treatments *A* - *C*.

	Estimate	Std. Error	t value	Pr(>|t|)
(Intercept – Treatment *A* - Sediments)	0.78658	0.12440	6.323	1.01e-05
Treatment *B* – Microplastics	-0.09299	0.17593	-0.529	0.604
Treatment *C* – Sediments and microplastics	0.03669	0.19272	0.190	0.851

Adjusted R^2= -0.09m, p-value = 0.776

Figure S6

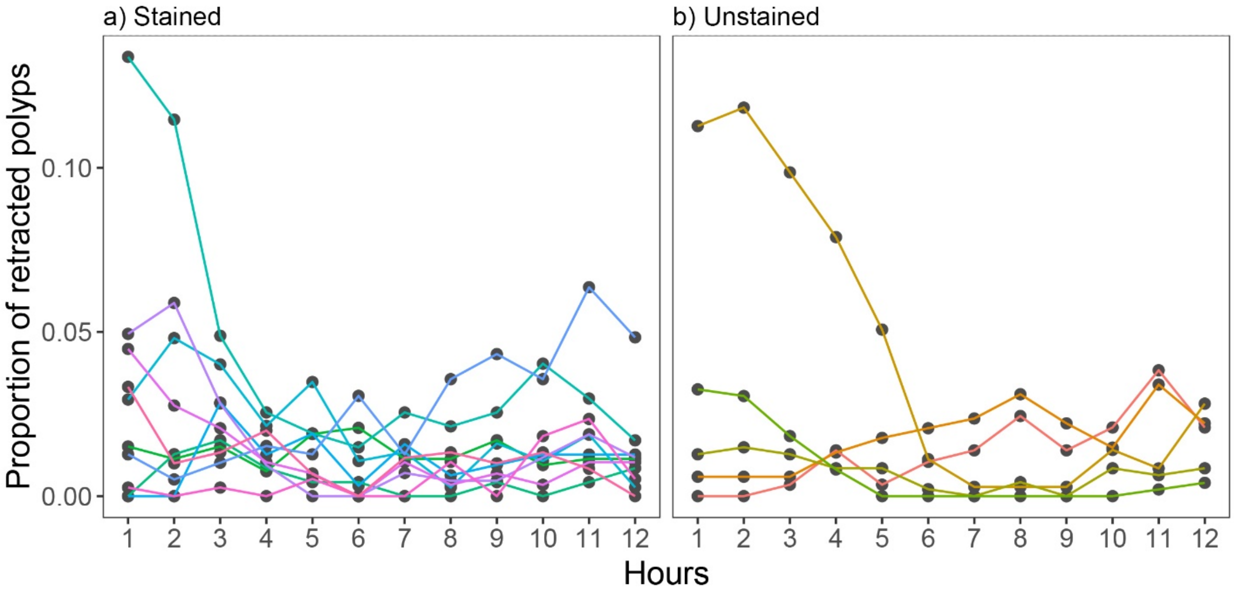

Fig. S6. Proportion of retracted polyps recorded hourly for 12 hours in a parallel experiment run on *Pocillopora damicornis* exposed to a) stained and b) unstained irregular PET microplastics. No significant differences were observed among hours or between the type of particles (Table S7).

Table S7

Table S7. Statistical outputs of a binomial generalised linear model (GLMER) fitted to data obtained during a parallel experiment exposing *P. damicornis* to *stained* (n = 9 fragments) and *unstained* (n = 5 fragments) irregular PET microplastics. The model tests whether the proportion of retracted polyps within colonies changed hourly for 12 hours, and whether this change was contingent on microplastic staining.

**Outputs of statistical model (Binomial GLMER)**				
Fixed effects	Estimate	SE	z-value	Pr (>|z|)
Intercept (Staining: *Stained*)	-4.02654	0.44157	-9.119	<2e-16
Hour	-0.05528	0.05919	-0.934	0.350
Staining: *Unstained*	0.11033	0.76183	0.145	0.885
hour:Staining: *Unstained*	-0.04062	0.10249	-0.396	0.692

Figure S7

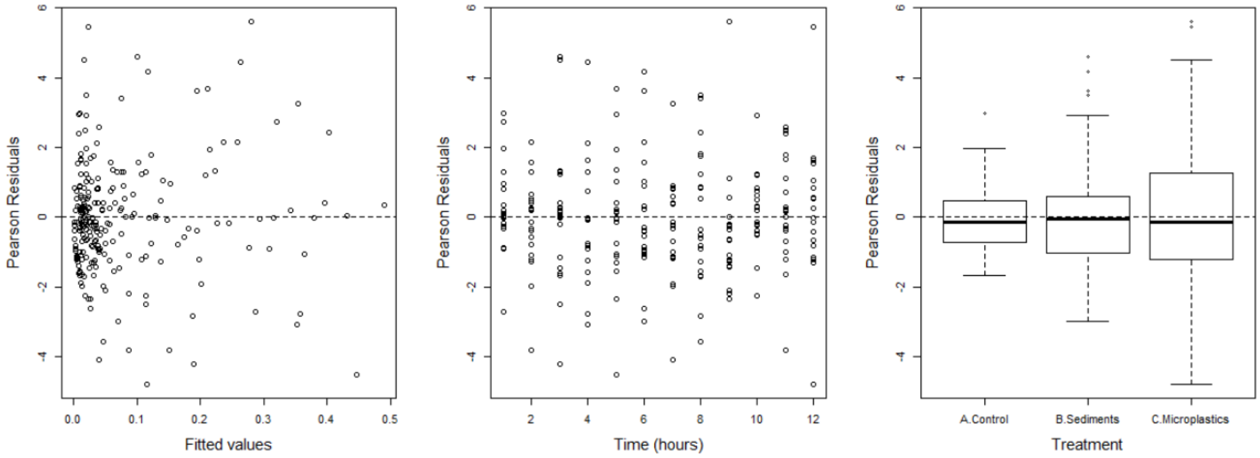

Fig. S7. Model validation plots for the GAMM with binomial distribution fitted to test whether the proportion of polyps retracted over time differed when exposed to control experimental conditions, sediments, and microplastics.

References cited

1. Hall, N. M., Berry, K. L. E., Rintoul, L. & Hoogenboom, M. O. Microplastic ingestion by scleractinian corals. *Mar. Biol.* **162**, 725–732 (2015).
2. Connors, E. J. Distribution and biological implications of plastic pollution on the fringing reef of Moorea , French Polynesia. *PeerJ* **5**, e3733 (2017).
3. Cai, M. *et al.* Lost but can't be neglected: Huge quantities of small microplastics hide in the South China Sea. *Sci. Total Environ.* **633**, 1206–1216 (2018).
4. Chapron, L. *et al.* Macro- and microplastics affect cold-water corals growth, feeding and behaviour. *Sci. Rep.* **8**, 1–8 (2018).
5. Cheang, C. C., Ma, Y. & Fok, L. Occurrence and composition of microplastics in the seabed sediments of the coral communities in proximity of a metropolitan area. *Int. J. Environ. Res. Public Health* **15**, (2018).
6. Cordova, M. R., Purwiyanto, A. I. S. & Suteja, Y. Abundance and characteristics of microplastics in the northern coastal waters of Surabaya, Indonesia. *Mar. Pollut. Bull.* **142**, 183–188 (2019).
7. Saliu, F. *et al.* Microplastic and charred microplastic in the Faafu Atoll, Maldives. *Mar. Pollut. Bull.* **136**, 464–471 (2018).
8. Jeyasanta, K. I., Patterson, J., Grimsditch, G. & Edward, J. K. P. Occurrence and characteristics of microplastics in the coral reef, sea grass and near shore habitats of Rameswaram Island, India. *Mar. Pollut. Bull.* **160**, 111674 (2020).
9. Zhu, L. *et al.* Microplastic pollution in North Yellow Sea, China: Observations on occurrence, distribution and identification. *Sci. Total Environ.* **636**, 20–29 (2018).
10. Huang, Y. *et al.* Distribution characteristics of microplastics in Zhubi Reef from South China Sea. *Environ. Pollut.* **255**, 113133 (2019).
11. Jensen, L. H., Motti, C. A., Garm, A. L., Tonin, H. & Kroon, F. J. Sources, distribution and fate of microfibres on the Great Barrier Reef, Australia. *Sci. Rep.* **9**, 1–15 (2019).
12. Saliu, F., Montano, S., Leoni, B., Lasagni, M. & Galli, P. Microplastics as a threat to coral

reef environments: Detection of phthalate esters in neuston and scleractinian corals from the Faafu Atoll, Maldives. *Mar. Pollut. Bull.* **142**, 234–241 (2019).
13. Zhang, L., Zhang, S., Wang, Y., Yu, K. & Li, R. The spatial distribution of microplastic in the sands of a coral reef island in the South China Sea: Comparisons of the fringing reef and atoll. *Sci. Total Environ.* **688**, 780–786 (2019).
14. Ding, J. *et al.* Microplastics in the Coral Reef Systems from Xisha Islands of South China Sea. *Environ. Sci. Technol.* 1–16 (2019) doi:10.1021/acs.est.9b01452.
15. Nie, H., Wang, J., Xu, K., Huang, Y. & Yan, M. Microplastic pollution in water and fish samples around Nanxun Reef in Nansha Islands, South China Sea. *Sci. Total Environ.* **696**, 134022 (2019).
16. Bucol, L. A. *et al.* Microplastics in marine sediments and rabbitfish (*Siganus fuscescens*) from selected coastal areas of Negros Oriental, Philippines. *Mar. Pollut. Bull.* **150**, 110685 (2020).
17. Patterson, J. & Immaculate Jeyasanta, K. Microplastic and heavy metal distributions in an Indian coral reef ecosystem. *Sci. Total Environ.* **744**, 140706 (2020).
18. Tan, F. *et al.* Microplastic pollution around remote uninhabited coral reefs of Nansha Islands, South China Sea. *Sci. Total Environ.* **725**, 138383 (2020).
19. Tang, J. *et al.* Differential enrichment and physiological impacts of ingested microplastics in scleractinian corals in situ. *J. Hazard. Mater.* **404**, 124205 (2021).
20. Allen, A. S., Seymour, A. C. & Rittschof, D. Chemoreception drives plastic consumption in a hard coral. *Mar. Pollut. Bull.* **124**, 198–205 (2017).
21. Reichert, J., Schellenberg, J., Schubert, P. & Wilke, T. Responses of reef building corals to microplastic exposure. *Environ. Pollut.* 1–6 (2017).
22. Hankins, C., Duffy, A. & Drisco, K. Scleractinian coral microplastic ingestion: Potential calcification effects, size limits, and retention. *Mar. Pollut. Bull.* **135**, 587–593 (2018).
23. Tang, J., Ni, X., Zhou, Z., Wang, L. & Lin, S. Acute microplastic exposure raises stress response and suppresses detoxification and immune capacities in the scleractinian coral Pocillopora damicornis. *Environ. Pollut.* **243**, 66–74 (2018).
24. Syakti, A. D. *et al.* Bleaching and necrosis of staghorn coral (Acropora formosa) in laboratory assays: Immediate impact of LDPE microplastics. *Chemosphere* **228**, 528–535 (2019).
25. Mouchi, V. *et al.* Long-term aquaria study suggests species-specific responses of two cold-water corals to macro-and microplastics exposure. *Environ. Pollut.* **253**, 322–329 (2019).
26. Rotjan, R. D. *et al.* Patterns, dynamics and consequences of microplastic ingestion by the temperate coral, *Astrangia poculata*. *Proc. R. Soc. B Biol. Sci.* **286**, 20190726 (2019).
27. Reichert, J., Arnold, A. L., Hoogenboom, M. O., Schubert, P. & Wilke, T. Impacts of microplastics on growth and health of hermatypic corals are species-specific. *Environ. Pollut.* **254**, 113074 (2019).
28. Martin, C., Corona, E., Mahadik, G. A. & Duarte, C. M. Adhesion to coral surface as a potential sink for marine microplastics. *Environ. Pollut.* **255**, 113281 (2019).
29. Axworthy, J. B. & Padilla-Gamiño, J. L. Microplastics ingestion and heterotrophy in thermally stressed corals. *Sci. Rep.* **9**, 1–8 (2019).
30. Berry, L., Epstein, H., Lewis, P., Hall, N. & Negri, A. Microplastic contamination has limited effects on coral fertilisation and larvae. *Diversity* **11**, 228 (2019).
31. Corona, E., Martin, C., Marasco, R. & Duarte, C. M. Passive and active removal of marine

microplastics by a mushroom Coral (*Danafungia scruposa*). *Front. Mar. Sci.* **7**, 1–9 (2020).
32. Feng, L. *et al.* Investigating the composition and distribution of microplastics surface biofilms in coral areas. *Chemosphere* **252**, 126565 (2020).
33. Lanctôt, C. M. *et al.* Physiological stress response of the scleractinian coral Stylophora pistillata exposed to polyethylene microplastics. *Environ. Pollut.* **263**, (2020).
34. Okubo, N., Tamura-Nakano, M. & Watanabe, T. Experimental observation of microplastics invading the endoderm of anthozoan polyps. *Mar. Environ. Res.* **162**, 105125 (2020).
35. Rocha, R. J. M. *et al.* Do microplastics affect the zoanthid *Zoanthus sociatus*? *Sci. Total Environ.* **713**, 136659 (2020).
36. Mendrik, F. M. *et al.* Species-specific impact of microplastics on coral physiology. *Environ. Pollut.* **269**, 116238 (2021).
37. Reichert, J. *et al.* Interactive effects of microplastic pollution and heat stress on reef-building corals. *Environ. Pollut.* **290**, 118010 (2021).
